# Green Synthesized Silver Nanoparticles from Biowaste for Rapid Dye Degradation: Experimental Investigation and Computational Mechanistic Insights

**DOI:** 10.3390/molecules30183738

**Published:** 2025-09-15

**Authors:** Tanakorn Wonglakhon, Areeya Chonsakon, Prawit Nuengmatcha, Benjawan Ninwong, Dirk Zahn, Yanisa Thepchuay

**Affiliations:** 1 Futuristic Science Research Center, School of Science, Walailak University, Nakhon Si Thammarat 80160, Thailand; tanakorn.wo@mail.wu.ac.th; 2 Research Center for Theoretical Simulation and Applied Research in Bioscience and Sensing, Walailak University, Nakhon Si Thammarat 80160, Thailand; 3 Center of Excellence in Nanomaterials Chemistry, Faculty of Science and Technology, Nakhon Si Thammarat Rajabhat University, Nakhon Si Thammarat 80280, Thailand; 4 Department of Chemistry, Faculty of Science and Technology, Nakhon Si Thammarat Rajabhat University, Nakhon Si Thammarat 80280, Thailand; 5 Lehrstuhl für Theoretische Chemie/Computer Chemie Centrum, Friedrich-Alexander-Universität Erlangen-Nürnberg, Nägelsbachstraße 25, 91052 Erlangen, Germany; 6 Flow Innovation-Research for Science and Technology Laboratories (Firstlabs), Bangkok 10400, Thailand

**Keywords:** Ag NPs, *Nypa fruticans*, dye degradation, molecular dynamics, density functional theory

## Abstract

Silver nanoparticles (Ag NPs) green-synthesized using *Nypa fruticans* fruit husk (NF) extract were applied as catalysts for the degradation of organic dyes in water for the first time. The synthesized Ag NPs, which were well-dispersed, highly stable, and small in size with an average diameter of ~4 nm, efficiently catalyzed the degradation of methyl orange (MO) in the presence of ${\mathrm{NaBH}}_{4}$, achieving complete degradation (>99%) within one minute under optimized conditions. The application to a commercial synthetic dye resulted in over 89% degradation within five minutes. To elucidate the degradation mechanism at the atomistic level, molecular dynamics (MD) simulations and density functional theory (DFT) calculations were employed. MD simulations revealed the adsorption behavior of MO on the Ag(111) surface. DFT calculations clarified the reaction pathway of MO degradation, identifying direct hydride transfer from ${\mathrm{BH}}_{4}^{-}$ to the azo group of MO as the rate-determining step, with the subsequent step influenced by the pH conditions. These findings illustrate the potential of NF extract in the green synthesis of catalytically active Ag NPs and contribute to understanding their role in dye degradation processes relevant to environmental remediation.

## 1. Introduction

Water pollution from dyes poses severe risks to both ecosystems and human health. Industrial and domestic activities contribute significantly to the release of these contaminants, which disrupt surface water chemistry and harm aquatic life. Annually, approximately 7 × 10^7^ tons of synthetic dyes are produced worldwide, with textile industries consuming over 10,000 tons [1]. The textile industry discharges large volumes of highly colored wastewater containing various synthetic dyes, including azo, reactive, and sulfide dyes [2]. However, even trace amounts of these dyes (e.g., 1 × 10^−3^ mg/L) are hazardous and can cause mutagenic and carcinogenic effects that impact the human nervous, digestive, renal, and hepatic systems [3]. 

In essence, effective degradation or removal of dyes is essential and urgently needed. Methods for detecting and removing organic pollutants are typically divided into chemical (e.g., reduction, photochemical degradation), physical (e.g., adsorption, filtration), and biological processes, with adsorption being a simple and commonly used approach [4,5]. In response to the limitations of traditional techniques, researchers are increasingly focusing on simpler and more cost-effective alternatives. One promising option is a colorimetric method using biologically synthesized nanoparticles, particularly silver nanoparticles (Ag NPs). These nanoparticles show potential for catalytically reducing dyes due to their unique properties, such as high surface area, strong catalytic activity, and the ability to change color when interacting with pollutants [6,7,8,9,10,11]. Furthermore, Ag NP-based methods are relatively easy to implement and less costly than more complex and expensive instruments, rendering Ag NPs one of the practical choices for environmental applications.

Ag NPs synthesized from various natural extracts, such as those from mango leaf [6], cauliflower waste [8], and *Epilobium parviflorum* green tea [7], have been shown to efficiently degrade hazardous organic dyes, and even detect heavy metal ions. In our recent study [12], Ag NPs were successfully synthesized using the *Nypa fruticans* fruit husk (NF) extract [13,14], which is a common palm species grown in Thailand, particularly in the south. Nipa palms have been used for various products such as syrup, sugar, and vinegar derived from the palm sap [15], leaving large quantities of nipa palm waste unemployed. This has inspired us to transform waste into valuable resources by exploring its potential for Ag NP synthesis. This biowaste-based synthesis adds value to waste materials and supports sustainable waste management by producing valuable nanomaterials, in line with the Sustainable Development Goals (SDGs) [16]. Our synthesized Ag NPs have already been shown to be highly selective toward Fe^2+^ metal ions [12]. 

While Ag NPs are known to act as electron relays that facilitate color changes and catalytic activity, in the present study, we present a combined experimental and theoretical analysis of the degradation of hazardous methyl orange (MO) dye. We thus extend the application perspectives of the Ag NPs synthesized using the NF extract, for the first time, to the efficient and eco-friendly degradation of organic dyes and provide mechanistic insights from molecular dynamics simulations and quantum chemical calculations, respectively. 

## 2. Results and Discussion

### 2.1. Optimized Conditions for the Synthesis of Ag NPs

In our previous study [12], we optimized the synthesis conditions of Ag NPs using NF extract by analyzing their surface plasmon resonance (SPR) bands at 400–425 nm [17] via UV–Vis spectroscopy. The optimal conditions were determined through systematic variations in the key reaction parameters: pH, Ag${\mathrm{NO}}_{3}$ concentration, NF extract concentration, volume ratio of NF extract to Ag${\mathrm{NO}}_{3}$, temperature, and reaction time. The highest SPR intensity with a narrow band, indicating well-dispersed Ag NPs, was achieved at pH 9.0. The optimal Ag${\mathrm{NO}}_{3}$ concentration was 1.0 mM, while 0.2% (*w*/*v*) NF extract was sufficient for complete reduction and stabilization. A volume ratio of NF extract to Ag${\mathrm{NO}}_{3}$ of 1:20 provided maximum yield. Temperature optimization revealed that 60 °C was appropriate, as higher temperatures led to precipitation. Lastly, a reaction time of 45 min was found to be optimal, as prolonged durations led to particle aggregation. These optimized conditions (Table 1) were established to ensure the formation of stable, uniformly distributed Ag NPs with controlled size and shape.

### 2.2. Characteristics and Stability of the Synthesized Ag NPs 

**Figure 1:**
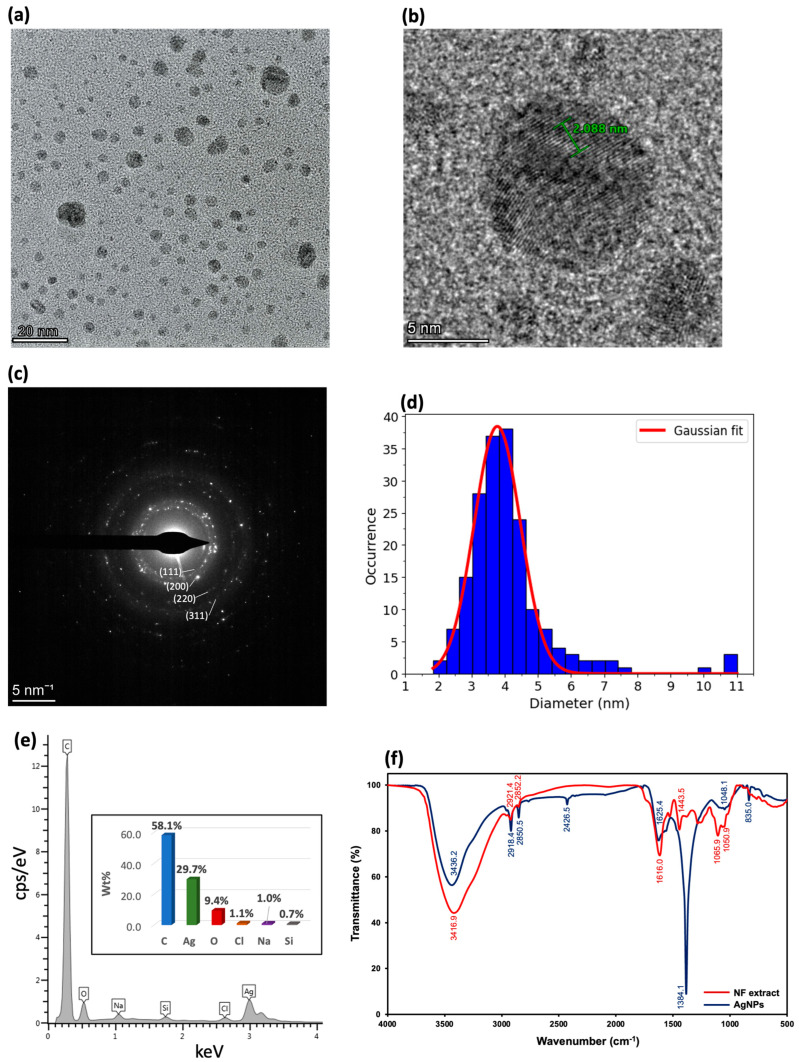
(**a**) TEM image of Ag NPs derived from the NF extract. (**b**) High-resolution TEM image highlighting the lattice fringes of the crystalline Ag NPs. (**c**) Selected area electron diffraction (SAED) pattern confirming the crystalline nature of the synthesized Ag NPs. (**d**) Size distribution profile of Ag NP diameters corresponding to the particles in (**a**). (**e**) EDX spectrum from SEM-EDX analysis showing elemental composition of the Ag NPs. (**f**) FT-IR spectra comparing the NF extract (red line) and Ag NPs synthesized using the NF extract (blue line). Reprinted/adapted from ref. [12], Copyright (2025), with permission from Elsevier.

As reported in our previous study [12], we established that NF extract-mediated Ag NPs exhibit stable, well-dispersed, highly crystalline structures with a mean size of ∼4 nm (Figure 1a–d). Elemental analysis via EDX confirmed the presence of Ag along with trace elements from the extract (Figure 1e). FT-IR spectra indicated strong interactions between phytochemicals and the Ag NP surface, particularly through O–H and aromatic groups (Figure 1f). The stability test showed that our prepared Ag NPs were stable for at least two weeks, showing potential for long-term storage. Theoretical studies using DFT and MD simulations revealed that phenolic compounds play a key role as reducing and stabilizing agents, showing a high affinity for the Ag(111) surface, which prevents uncontrolled aggregation. At the optimal pH of 9, deprotonation of phenolic groups resulted in negatively charged, phenolic compound-functionalized Ag NPs, as evidenced by a zeta potential of −41.9 mV, which contributed to enhanced colloidal stability by preventing aggregation. Building on these findings, the present study extends the application of the Ag NPs synthesized using the same synthesis conditions to the degradation of methyl orange dye, which is a toxic and non-biodegradable azo dye, as illustrated in subsequent sections. To confirm batch reproducibility, the newly synthesized Ag NPs were re-characterized by UV–Vis spectrophotometry. The spectrum showed a characteristic SPR band at 424 nm with a peak absorbance of 1.6415, closely matching our previous batch (1.6493) [12]; the peak absorbances differ by less than 0.5%. Figure S1 displays the raw spectral overlay: the plasmon peak position and the peak profile are essentially identical; any differences are predominantly on the long-wavelength tail (λ > 450 nm). These results verify that the Ag NPs used here were distinct yet reproducible under the same synthesis conditions.

### 2.3. Catalytic Reduction of Methyl Orange Dye

To illustrate the application of our synthesized Ag NPs, we assess the function of the synthesized Ag NPs to act as the catalysts for the dye degradation. For this, the catalytic activity of the synthesized Ag NPs for degrading MO was evaluated in the presence of ${\mathrm{NaBH}}_{4}$. In aqueous medium, the azo (–N=N–) group of the MO shows a characteristic absorption peak at 464 nm. Therefore, we focus on the spectrum change at this wavelength, which shows the maximum absorbance at 464 nm, to track the dye degradation. 

**Figure 2:**
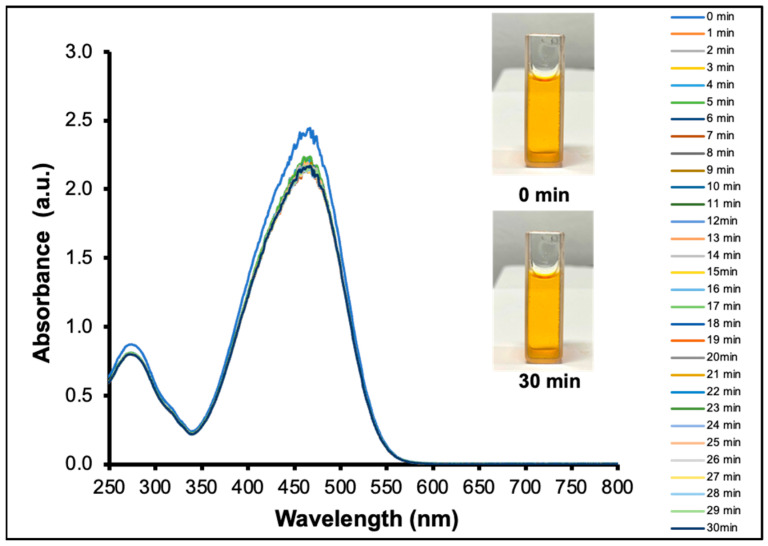
Methyl orange dye degradation in the presence of ${\mathrm{NaBH}}_{4}$ without Ag NPs. After 30 min, the solution color virtually remained unchanged.

Firstly, the MO dyes were found to degrade very slowly in the absence of Ag NPs as indicated by the very slow decrease in the absorbance spectrum as a function of time (Figure 2). This may be hindered by the electrostatic repulsion between the negatively charged borohydride ion (${\mathrm{BH}}_{4}^{-}$) and the negatively charged MO dye (an anionic dye), making the reduction reaction difficult to surpass the energy barrier. Upon the introduction of the Ag NPs to the system, the MO degradation is expected to proceed faster since the Ag NPs act as the electron relay to facilitate the ${\mathrm{NaBH}}_{4}$ to reduce the MO dyes.

**Figure 3:**
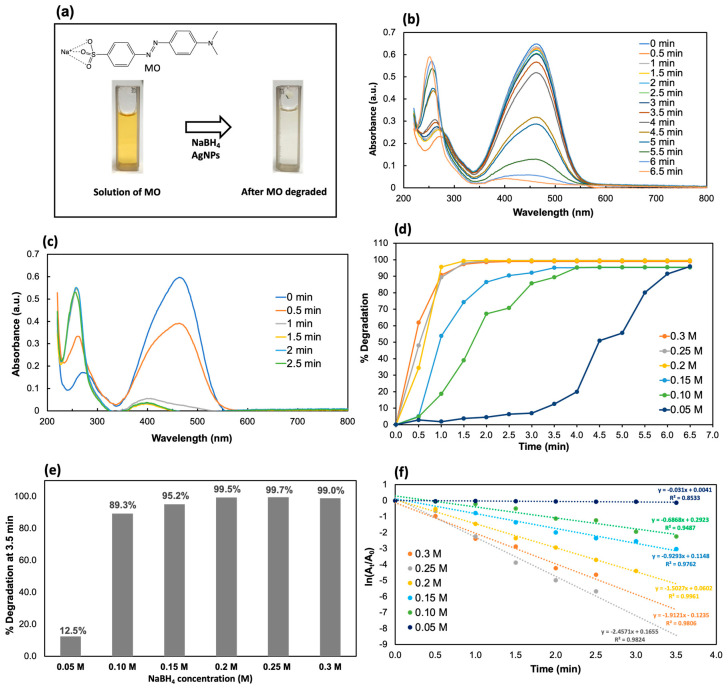
(**a**) Illustration of the decolorization of a yellow MO solution by the synthesized Ag NPs in the presence of ${\mathrm{NaBH}}_{4}$. (**b**) UV–Vis spectral changes in the MO degradation by the synthesized Ag NPs at 0.05 M ${\mathrm{NaBH}}_{4}$. (**c**) UV–Vis spectral changes in the MO degradation by the synthesized Ag NPs at 0.20 M ${\mathrm{NaBH}}_{4}$. (**d**) Percent degradation of MO dye as a function of time at 0.05, 0.10, 0.15, 0.20, 0.25, and 0.30 M ${\mathrm{NaBH}}_{4}$, respectively. (**e**) Percent degradation of MO dye at the first 3.5 min as a function of ${\mathrm{NaBH}}_{4}$ concentration. (**f**) Pseudo-first-order kinetics of MO degradation at different ${\mathrm{NaBH}}_{4}$ concentrations.

To find the suitable concentration of ${\mathrm{NaBH}}_{4}$ for the MO degradation, we studied six concentrations of 0.5 mL ${\mathrm{NaBH}}_{4}$ (i.e., 0.05, 0.10, 0.15, 0.20, 0.25, and 0.30 M) while keeping the Ag NP solution at 50 μL and the MO solution at 2.5 mL (0.1 mM). The catalytic efficiency was then assessed as the percent of MO degradation as a function of time. Figure 3a shows the initial MO solution and the resulting decolorized solution after degradation. As shown in Figure 3d,e, at a given time, the degradation percentages of MO increased significantly with the increase in the ${\mathrm{NaBH}}_{4}$ concentration. However, upon increasing the concentration of ${\mathrm{NaBH}}_{4}$ to 0.2 M, the MO was effectively degraded over 99%, which is not significantly different when the ${\mathrm{NaBH}}_{4}$ concentration was increased to 0.25 M (Figure 3e). This corresponds to a rapid decrease in the absorbance spectrum within 2.5 min at 0.20 M ${\mathrm{NaBH}}_{4}$ (Figure 3c vs. Figure 3b). In addition, when the ${\mathrm{NaBH}}_{4}$ concentration increases to 0.3 M, we observed a decrease in the degradation percentage and the rate constant. Therefore, 0.20 M ${\mathrm{NaBH}}_{4}$ is already enough to reduce the MO dyes on the Ag NPs. As shown in Figure 3f, the MO degradation follows the pseudo-first-order kinetics, ln(A_t_/A_0_) = −kt, where k is the degradation rate constant and A_0_ and A_t_ are the absorbances at t = 0 and time t, respectively. From the plot of ln(A_t_/A_0_) as a function of time (Figure 3f), a rate constant for each ${\mathrm{NaBH}}_{4}$ concentration was derived and is shown in Table 2. It can be concluded that the rate constant increased with the ${\mathrm{NaBH}}_{4}$ concentration up to 0.25 M but declined at 0.30 M. Because >99% MO degradation was achieved within 2.5 min at 0.20 M, we selected 0.20 M ${\mathrm{NaBH}}_{4}$ as the working concentration. 

**Figure 4:**
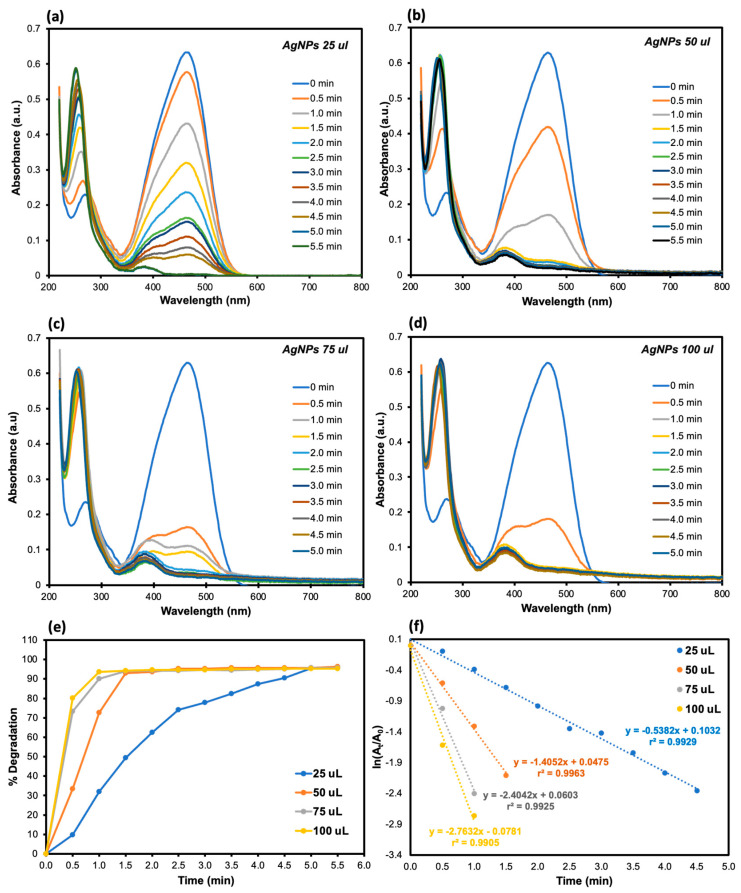
UV–Vis spectral changes in the MO degradation by the synthesized Ag NPs in the presence of 0.20 M ${\mathrm{NaBH}}_{4}$ at (**a**) 25 μL, (**b**) 50 μL, (**c**) 75 μL, and (**d**) 100 μL of 0.20 M ${\mathrm{NaBH}}_{4}$, respectively. (**e**) Percent degradation of MO dye as a function of time at 25 μL, 50 μL, 75 μL, and 100 μL of 0.20 M ${\mathrm{NaBH}}_{4}$, respectively. (**f**) Corresponding pseudo-first-order kinetics of MO degradation at different ${\mathrm{NaBH}}_{4}$ concentrations.

The effect of the amount of Ag NP catalyst on the catalytic reduction of MO azo dye was further investigated by varying the volume of the Ag NP catalyst from 25 μL to 100 μL at a fixed concentration of ${\mathrm{NaBH}}_{4}$ (0.2 M) and MO dye (0.1 mM). The reduction process was then monitored by the decrease in the absorbance peak at 465 nm and simultaneous increase in the absorbance at 250 nm (Figure 4). In analogy to the above study, the pseudo-first-order kinetics was used to assess the catalytic performance of Ag NPs. When the Ag NP volume increases from 25 to 100 μL, the rate constant was found to increase from 0.523 to 2.763 min^−1^ while the time for the complete MO degradation decreased from 4.5 to just 1 min (Table 3). At 50 μL, the MO degradation percentage already reached 95%. To ensure economical application of Ag NPs, we therefore chose 50 μL Ag NPs. 

### 2.4. Comparison with Other Studies

A comparison with other studies on using green-synthesized Ag NPs to degrade the MO dye in the presence of ${\mathrm{NaBH}}_{4}$ (from 2014 to the present) is summarized in Table 4. For a fair kinetic comparison, Table 4 excludes non-green syntheses and non-Ag NP catalysts for dye degradation (e.g., peroxymonosulfate (PMS)/Fenton-like oxidation [18,19]). As can be observed, the reported kinetic parameters vary considerably depending on factors such as the source of nanoparticle synthesis and catalyst size and morphology, including the Ag NP and ${\mathrm{NaBH}}_{4}$ concentrations. Compared to these studies, our Ag NPs synthesized using NF extract achieve more than 99% MO degradation within one minute and exhibit among the higher rate constant and shorter reduction time reported. This superior catalytic performance may be attributed to the small particle size and good dispersion of the Ag NPs in solution, which facilitate the interactions between MO dye molecules and ${\mathrm{NaBH}}_{4}$. Encouraged by these promising results, we extended the application of our Ag NPs to the degradation of a commercially available synthetic dye obtained from a local market in Thailand, as described in the following section.

### 2.5. Catalytic Reduction of Commercial Synthetic Dye for Cotton Fabrics

**Figure 5:**
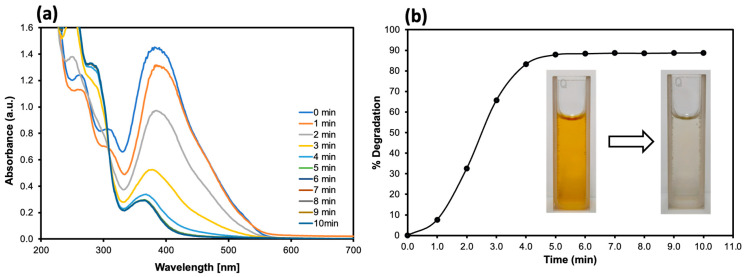
(**a**) UV–Vis spectral changes in the degradation of the commercial synthetic dye by the synthesized Ag NPs. (**b**) Percent degradation of the commercial synthetic dye. As can be seen, the MO degradation reached 89% within only 5 min.

To demonstrate the effectiveness of our synthesized Ag NPs, we investigated the catalytic reduction of a commercial synthetic dye, with direct deep yellow. Although this dye lacks a unique identification code or specific chemical name, it mainly contains azo, phenyl, and sulfonic acid groups and is widely used for coloring cotton fabrics in Thailand. The experiment was carried out at room temperature by mixing 2.5 mL of 1000 ppm synthetic dye (prepared with deionized water) and 0.5 mL of 0.2 M ${\mathrm{NaBH}}_{4}$ with 50 μL of Ag NP solution. At the beginning, the color solution appeared a deep yellow color. As the reaction progressed, the deep yellow solution decolorized, accompanied by a rapid decrease in the absorbance at 465 nm (Figure 5a), yielding more than 89% degradation within only 5 min (Figure 5b). In real water (non-potable tap water), degradation was ~83% at 5 min and reached ~86% by 6 min for two independent samples (Figure S2), consistent with a modest matrix effect, while maintaining high catalytic performance. Therefore, these results indicate that Ag NPs synthesized using NF extract are promising candidates for the catalytic treatment of wastewater containing non-biodegradable azo dye.

### 2.6. Insights into the Degradation Mechanism of Methyl Orange via Molecular Simulations

#### 2.6.1. MO Dye Adsorption Mechanism via MD Simulations 

While several studies have explored the use of Ag NPs in the degradation of MO dye (as shown in Table 4), they commonly suggest that Ag NPs act as electron relays that facilitate the degradation process. However, the underlying mechanisms—particularly at the atomistic level—remain poorly understood. From the molecular dynamics perspective, which is well suited for investigating adsorption behavior, no prior simulations have specifically examined the adsorption of MO onto silver nanoparticle surfaces.

On other materials, a few theoretical studies have employed MD simulations to investigate the adsorption behavior of MO dye on solid surfaces. For example, Fahimirad et al. conducted combined experimental and theoretical work on MO photodegradation using Au-ZnO catalysts [56]. Their theoretical study employed the Monte Carlo method, which is similar in concept to MD, to model MO adsorption on Au-ZnO(111) with 10 water molecules. Despite the arbitrary placement of 10 Au atoms on the ZnO(111) surface, the MO molecule was observed to lie flat on the surface. Similarly, Boumya et al. used MD simulations to study MO adsorption on the (110) surface of various metal chlorides and concluded that the dye adsorbs via van der Waals interactions, with a separation distance of over 3.0 Å [57].

**Figure 6:**
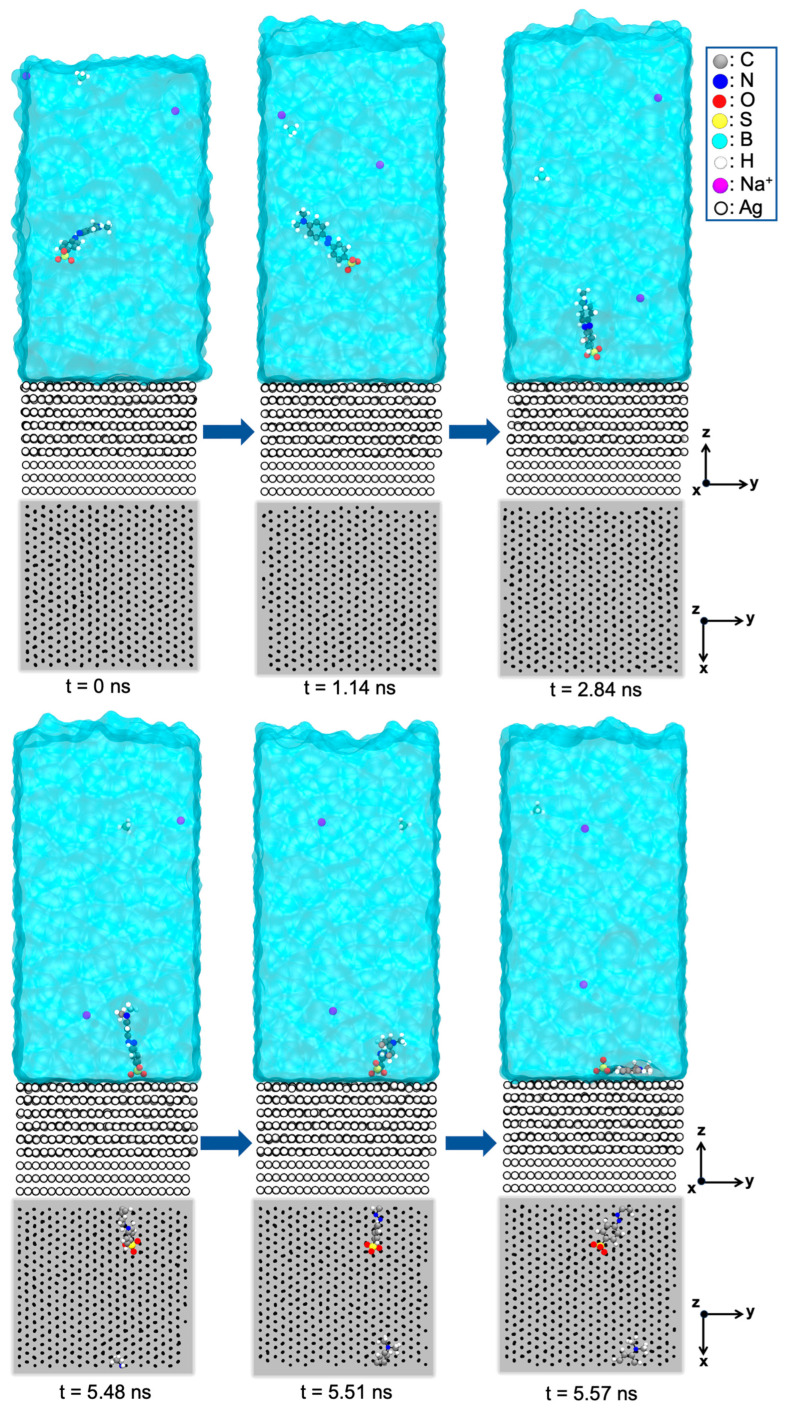
Snapshots from MD simulations over the course of 0 to 5.57 ns for the Ag–water interface model containing 2000 $H_{2}O$ molecules, 1 MO dye, and 1 $\mathrm{Na}{\mathrm{BH}}_{4}$ molecule, respectively. The time series is selected to illustrate the adsorption process of MO onto the Ag(111) surface. In parallel to the lateral perspective, we also show a top view of all atoms, except those of water, within 6 Å distance from the Ag(111) surface.

To investigate MO adsorption on the Ag(111) surface, we performed MD simulations of an extended Ag–water interface featuring 1 anionic MO molecule (${\mathrm{MO}}^{-}$), 2 ${\mathrm{Na}}^{+}$, 1 ${\mathrm{BH}}_{4}^{-}$, and 2000 water molecules. After energy minimization, we performed an MD run of 8 ns at 300 K. Within the first 3 ns, the sulfonate group of ${\mathrm{MO}}^{-}$ migrated to the Ag(111) surface, and by 6 ns, the molecule adopted a flat orientation on the surface (Figure 6). Upon further prolongation of the MD simulation, no desorption of ${\mathrm{MO}}^{-}$ was observed. These findings support the commonly proposed mechanism in which Ag NPs facilitate electron transfer (from ${\mathrm{BH}}_{4}^{-}$) by forming stable interactions with dye (MO) molecules.

**Figure 7:**
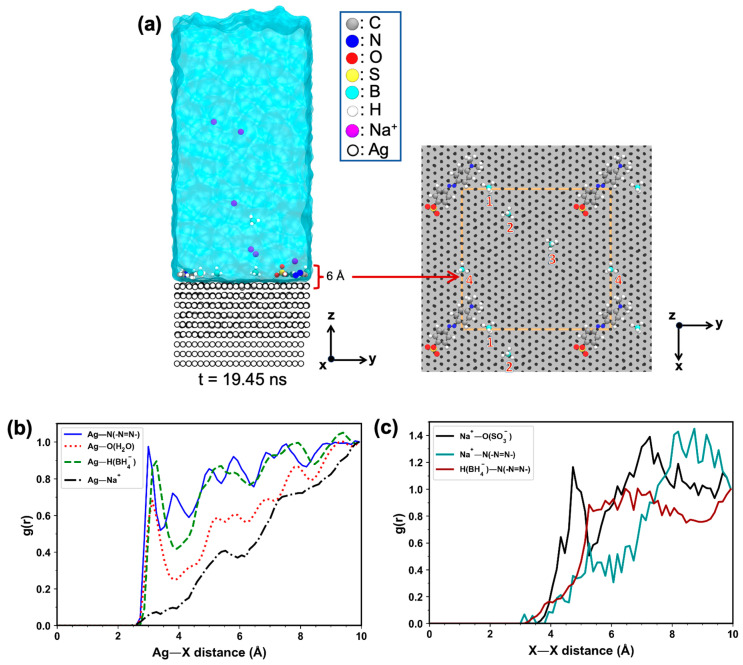
(**a**) Snapshot from the MD simulation at 19.45 ns showing the adsorption of four ${\mathrm{BH}}_{4}^{-}$ ions onto the Ag(111) surface. The right panel presents the top view of atoms, except $H_{2}O$, within 6 Å above the Ag(111) surface. The numbers 1–4 denote the four ${\mathrm{BH}}_{4}^{-}$ ions, while the orange rectangle outlines the simulation cell (periodic in x and y; free along z). (**b**) Normalized radial distribution functions, g(r), between Ag atoms and various interacting species: N atoms of the azo group in MO dye (blue solid curve), O atoms of water (red dotted curve), H atoms of ${\mathrm{BH}}_{4}^{-}$ (green dashed curve), and ${\mathrm{Na}}^{+}$ ions (black dash–dotted curve). The first peak maxima of g(r) for the Ag—N(–N=N–), Ag—O($H_{2}O$), Ag—H(${\mathrm{BH}}_{4}^{-}$), and Ag—${\mathrm{Na}}^{+}$ pairs occur at 3.00 Å, 3.13 Å, 3.27 Å, and ~3.20 Å (barely observed), respectively. Data were sampled from the final 4 ns of the simulation. (**c**) Normalized g(r) for selected ion–dye atom pairs: ${\mathrm{Na}}^{+}$—O of the sulfonate group (black solid line), ${\mathrm{Na}}^{+}$—N of the azo group (cyan solid curve), and H(${\mathrm{BH}}_{4}^{-}$)—N of the azo group (brown solid curve). The first peak maxima for the ${\mathrm{Na}}^{+}$—O(${\mathrm{SO}}_{3}^{-}$), ${\mathrm{Na}}^{+}$—N(–N=N–), and H(${\mathrm{BH}}_{4}^{-}$)—N(–N=N–) pairs are located at ~4.70 Å and ~3.00 Å (the latter two barely observed). Data were taken from the same 4 ns runs as in (**b**).

To investigate the adsorption behavior of ${\mathrm{BH}}_{4}^{-}$ ions on the Ag(111) surface, we introduced additional four ${\mathrm{BH}}_{4}^{-}$ ions and four ${\mathrm{Na}}^{+}$ counterions into the final configuration from the previous MD simulation. As shown in Figure 7a (snapshot at 19.45 ns), up to four ${\mathrm{BH}}_{4}^{-}$ ions were adsorbed onto the Ag(111) surface and were observed to diffuse laterally along the surface, indicating favorable interactions between ${\mathrm{BH}}_{4}^{-}$ and the Ag(111) surface.

To further characterize the adsorption of different species, radial distribution function (RDF) analysis was performed (Figure 7b,c). The RDF was normalized such that g(r) = 1 at r = 10 Å to allow direct comparison between different species. This distance was chosen because the number density of water was found to converge at around 10 Å (Figure S3), making it an appropriate reference point. The RDF of the nitrogen atom in the azo group of ${\mathrm{MO}}^{-}$ relative to the surface Ag atoms exhibited a sharp peak at 3.00 Å (Figure 7b, blue solid curve). In turn, Ag–O contacts with the oxygen atoms of nearby water molecules displayed a peak at a slightly greater distance of 3.13 Å (Figure 7b, red dashed curve).

For ${\mathrm{BH}}_{4}^{-}$ ions, the RDF of hydride ($H^{-}$) atoms showed a pronounced peak at 3.27 Å from the Ag(111) surface (Figure 7b, green dashed curve), indicating association via van der Waals interactions. In contrast, ${\mathrm{Na}}^{+}$ ions showed only occasional and transient contact to the Ag(111) surface, with only a weak RDF peak appearing at ~3.20 Å (Figure 7b, black dash-dotted curve). Indeed, ${\mathrm{Na}}^{+}$ remains predominantly solvated in water rather than binding to the Ag surface.

We furthermore observed an ${\mathrm{Na}}^{+}$ ion transiently approaches the sulfonate group of ${\mathrm{MO}}^{-}$, as indicated by a pronounced RDF peak at a relatively large distance of ~4.70 Å (Figure 7c, black solid curve), arguably acting as a diffuse positive charge to neutralize the dye’s negative charge, rather than forming a direct, stable coordination. The residence time of ${\mathrm{Na}}^{+}$ ion within 5 Å of the oxygen atoms of the sulfonate group was found to be only 5.4 ps (Figure S4), indicating a short-lived interaction. RDF analysis also confirmed negligible interaction between ${\mathrm{Na}}^{+}$ and the azo group of ${\mathrm{MO}}^{-}$ (Figure 7c, cyan solid curve).

Similarly, ${\mathrm{BH}}_{4}^{-}$ ions exhibited a low probability of approaching the azo group of ${\mathrm{MO}}^{-}$ at ~3.00 Å (Figure 7c, brown solid curve), indicating limited direct interaction. This likely reflects the Coulomb repulsion that prevents ${\mathrm{BH}}_{4}^{-}$ from directly reacting with the azo group of ${\mathrm{MO}}^{-}$, in contrast to the favorable electrostatic attraction between ${\mathrm{Na}}^{+}$ and the sulfonate group. Overall, these MD simulation results support the general view of the MO degradation mechanism, in which Ag NPs simultaneously interact with both ${\mathrm{MO}}^{-}$ and ${\mathrm{BH}}_{4}^{-}$. In the absence of Ag NPs, these solute species would remain dispersed in solution, preventing effective electron transfer from ${\mathrm{BH}}_{4}^{-}$ to ${\mathrm{MO}}^{-}$. Thus, Ag NPs facilitate electron transfer without requiring direct contact between the reducing agent and the dye molecule.

#### 2.6.2. Insights into the MO Degradation Mechanisms via DFT Calculations 

While the MD simulations provided atomic-level insights into the adsorption behavior of MO dye on the Ag(111) surface, they could not capture the electronic details of the degradation reaction mechanism. To address the electron transfer between the two reactants, we therefore employed DFT calculations to investigate the possible reaction pathways underlying MO degradation.

**Figure 8:**
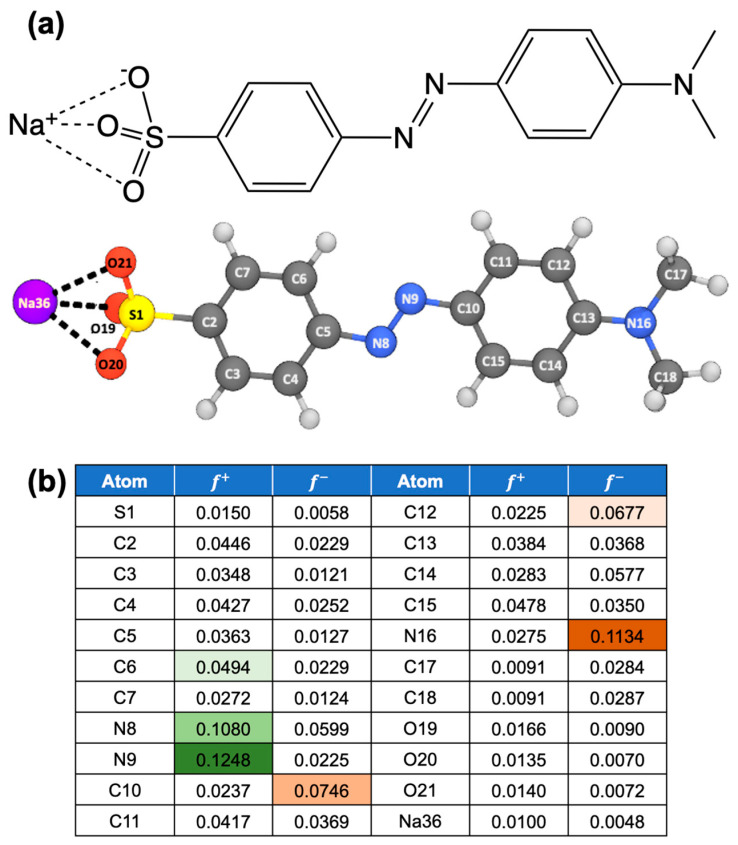
(**a**) Optimized geometry of a methyl orange dye with atom numbering. (**b**) A table of condensed Fukui index values ($f^{+}$ and $f^{-}$) of each atom of the optimized MO dye shown in (**a**). The darkest green color indicates the highest $f^{+}$ value, whilst the darkest orange color refers to the highest $f^{-}$ value.

As a first step, we aimed to identify the most reactive sites on the MO dye susceptible to the interaction with ${\mathrm{BH}}_{4}^{-}$. For this, condensed Fukui indices ($f^{+}$ and $f^{-}$) were calculated for each atom of the optimized MO geometry (Figure 8a,b). The Fukui function is a well-established theoretical tool for predicting site-specific chemical reactivity at the atomic level, where a high $f^{+}$ value indicates a site favorable for nucleophilic attack (electron donation), and a high $f^{-}$ value corresponds to electrophilic susceptibility (electron acceptance) [58,59,60]. 

The analysis revealed that the N9 atom of the azo group possesses the highest $f^{+}$ value (Figure 8b), suggesting it as the primary site for nucleophilic hydride attack by ${\mathrm{BH}}_{4}^{-}$. This hydride addition is expected to initiate electron delocalization within the azo group, with the neighboring N8 atom serving as an electron acceptor. Additionally, the C6 atom on the aromatic ring emerged as a secondary possible site for nucleophilic attack, though with lower reactivity than N9. On the other hand, the N16 atom exhibited the highest $f^{-}$ value, indicating its propensity to accept a proton ($H^{+}$), potentially forming a protonated amine H–N${\left({\mathrm{CH}}_{3}\right)}_{2}^{+}$ moiety. However, this protonation does not directly contribute to the azo bond cleavage or degradation of MO. Overall, the condensed Fukui index analysis provides a valuable foundation for our subsequent DFT-based mechanistic study of MO degradation.

***(A) First MO degradation***. Based on the condensed Fukui index values, we investigated the initial step of MO degradation using DFT calculations within a continuum water solvent model. Taking the free energy of the ${\mathrm{MO}}^{-}$ anion as the reference, the association of a ${\mathrm{Na}}^{+}$ ion with the sulfonyl group of ${\mathrm{MO}}^{-}$ slightly lowers the free energy by 0.6 kcal/mol. This suggests that ${\mathrm{Na}}^{+}$ remains weakly bound and can easily dissociate, in agreement with the calculated residence time of 5.4 ps from the above MD simulations.

**Figure 9:**
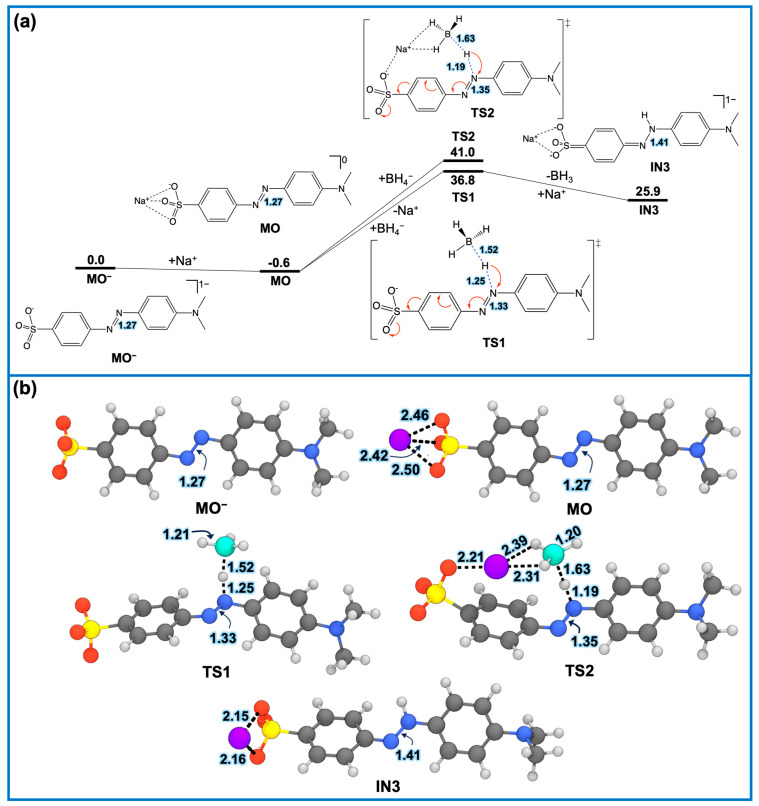
(**a**) Relative free-energy profile (in kcal/mol) for the first step of MO degradation via (i) a direct hydride transfer (TS1) and (ii) ${\mathrm{Na}}^{+}$-mediated hydride transfer (TS2) from ${\mathrm{BH}}_{4}^{-}$ to N9 atom of the azo group. The red curved arrows indicate electron flow. (**b**) Three-dimensional-optimized structures belong to those in (**a**), with distance labels in Å. Cartesian coordinates of all intermediates and transition states are provided in the Supporting Information.

The subsequent hydride attack from ${\mathrm{BH}}_{4}^{-}$ was explored via two possible pathways: (i) direct hydride transfer (TS1), defined by an imaginary frequency of −774.3 cm^−1^, and (ii) ${\mathrm{Na}}^{+}$-mediated hydride transfer (TS2), with an imaginary frequency of −673.0 cm^−1^, to the N9 atom of the azo group (Figure 9a). The direct hydride transfer proceeds with a lower activation energy (+37.4 kcal/mol) compared to the ${\mathrm{Na}}^{+}$-mediated pathway (+41.6 kcal/mol). Upon reaching the respective transition states (TS1 and TS2), the N–N bond length of the azo group increases from 1.27 Å to 1.33 Å (TS1) and 1.35 Å (TS2), indicating bond weakening (Figure 9b).

The lower activation energy for the direct hydride transfer (TS1) may be attributed to the higher aromaticity of the benzene ring attached to the ${\mathrm{SO}}_{3}^{-}$ group, compared to TS2. Aromaticity in this study is qualitatively assessed by analyzing deviations in C–C bond lengths and the S1–C2–C5 angle (see atom numbering in Figure 8a) relative to the unperturbed ${\mathrm{MO}}^{-}$ structure. The average C–C bond length deviation in TS2 is 0.9%, slightly larger than that of TS1 (0.5%). Moreover, the S1–C2–C5 angle in TS2 deviates by 11.4% from ${\mathrm{MO}}^{-}$, whereas in TS1, the deviation is only 0.9%. These structural distortions in TS2 are not sufficiently compensated by the greater charge transfer from ${\mathrm{BH}}_{4}^{-}$ to N9 (−0.807e for TS2 vs. −0.711e for TS1, based on natural population charge analysis).

It is worth noting that when ${\mathrm{Na}}^{+}$ was explicitly positioned at the sulfonyl group of ${\mathrm{MO}}^{-}$ and the direct hydride transfer transition state was sought, the optimization consistently converged to TS2, despite several attempts. After hydride transfer from ${\mathrm{BH}}_{4}^{-}$ to the N9 atom, the resulting intermediate (IN3) exhibits a relative free energy of +25.9 kcal/mol. The N–N distance of the azo group in IN3 further elongates to 1.41 Å, indicating significant bond weakening, which makes the molecule susceptible to further degradation. These findings suggest that while ${\mathrm{Na}}^{+}$ stabilizes charge in the system, its presence at a high concentration could inhibit MO degradation. This aligns with experimental observations showing that at higher $\mathrm{Na}{\mathrm{BH}}_{4}$ concentrations (~0.3 M), the apparent rate constant tends to decrease (see Table 2).

We also investigated the influence of a Ag atom by associating it with the azo group, as suggested by the MD simulations. This pathway exhibited an energy barrier of +38.3 kcal/mol, slightly higher than that of the direct hydride transfer (TS1) (Figure S5). These results imply that under the current model, Ag NPs primarily act as scaffolds to facilitate the proximity of MO and $\mathrm{Na}{\mathrm{BH}}_{4}$, rather than directly participating in the electron transfer process during this step. 

**Figure 10:**
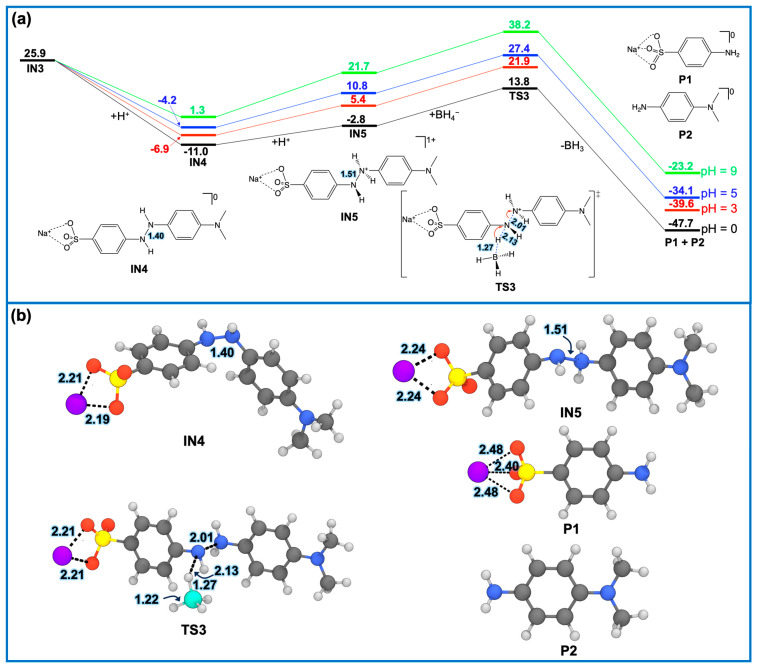
(**a**) Relative free-energy profile (in kcal/mol) for the second step of MO degradation. Since this step involves proton transfer processes, the relative free energies are corrected to pH values of 3 (red line), 5 (blue line), and 9 (green line), as described in the main text. This free-energy adjustment reveals the influence of pH on the energetics and mechanism of MO degradation. The red curved arrows indicate electron flow. (**b**) Three-dimensional-optimized structures belong to those in (**a**), with distance labels in Å. Cartesian coordinates of all intermediates and transition states are provided in the Supporting Information.

***(B) Second MO degradation***. The second step of MO degradation involves proton transfer processes that complete the breakdown of the MO molecule. To account for the effect of pH, the relative free energies (Figure 10a) were corrected to pH 3, 5, and 9 using the approach previously employed [61]. This allows a direct examination of how pH influences the energetics of the degradation pathway. Based on the optimal synthesis conditions for Ag NPs, the system pH (before the introduction of MO dye) is thus expected to be around 9. However, for the MO solution itself, the orangish hue suggests an acidic environment with a pH in the range of 3.1–4.4 [62]. Therefore, when the Ag NP dispersion and MO solution are mixed, the final pH is expected to be lower than 9.

Without pH correction (i.e., treating [$H^{+}$] = 1 M, pH = 0; black line in Figure 10a), following the formation of the intermediate IN3, this negatively charged species readily accepts a proton from the solution to form IN4 (a hydrazine-like structure) with a slightly reduced free energy of −11.0 kcal/mol. Subsequent protonation yields IN5, which has a slightly higher free energy of −2.8 kcal/mol. The N–N bond length in IN5 elongates to 1.51 Å (Figure 10b), indicating further bond weakening and susceptibility to cleavage. The intermediate IN5 then interacts with ${\mathrm{BH}}_{4}^{-}$ to undergo hydride transfer via TS3, with a low imaginary frequency of −267.1 cm^−1^, exhibiting a relative free-energy barrier of +24.8 kcal/mol, which is notably lower than that of the first step (+37.4 kcal/mol).

**Figure 11:**
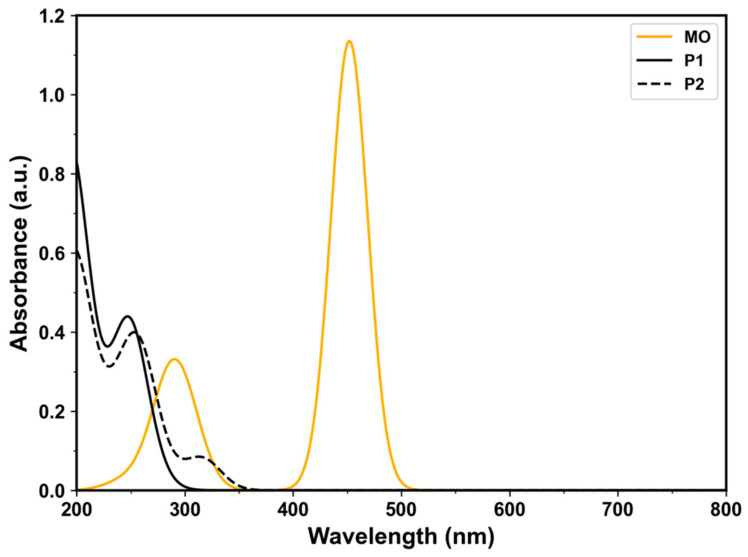
Simulated UV–Vis spectra obtained from TD-DFT calculations. The spectra compare methyl orange (MO, orange line), structure P1 (sodium 4-aminobenzenesulfonate, solid black line), and structure P2 (*N*,*N*-dimethyl-*p*-phenylenediamine, dashed black line).

This hydride transfer yields the degradation products P1 (sodium 4-aminobenzenesulfonate) and P2 (*N*,*N*-dimethyl-*p*-phenylenediamine). These products correlate with the observed increase in the UV–Vis absorbance at 250 nm (Figure 3 and Figure 4) [63]. To confirm this, we conducted TD-DFT calculations to predict the UV–Vis spectra of MO, P1, and P2. As shown in Figure 11, the predicted spectra agree well with the experimental spectra in Figure 3 and Figure 4, both in the peak positions and the spectral shifts observed during MO degradation.

At slightly higher pH values of 3 and 5, the energy barriers from IN4 to TS3 increase to +28.8 and +31.6 kcal/mol, respectively, though they remain lower than the barrier observed in the first step (Figure 10a vs. Figure 9a). However, at a basic pH of 9 (Figure 10a, green line), the energy barrier increases significantly to approximately 37.0 kcal/mol, comparable to that of the first step (+37.4 kcal/mol). Therefore, at basic pH (>9), the second hydride transfer becomes the rate-determining step. This suggests that MO degradation efficiency decreases at a higher pH, which is consistent with the findings of Kgatle et al. [64], who observed a similar pH-dependent degradation trend using trimetallic Fe/Cu/Ag nanoparticles. This aspect warrants further systematic investigation.

In conclusion, the present DFT study provides detailed insights into the mechanism of MO degradation. At low to neutral pH, the first step—direct hydride transfer via TS1—remains the rate-determining step. However, at a higher pH (>9), the second step involving proton transfer is energetically less favorable and thus becomes the rate-determining step. These theoretical findings are consistent with experimental observations and highlight the critical influence of pH on the degradation efficiency of MO dye.

## 3. Materials and Methods

### 3.1. Chemicals and Reagents

All chemicals employed were analytical grade. Silver nitrate (Ag${\mathrm{NO}}_{3}$) purchased from Sigma-Aldrich (Darmstadt, Germany), sodium borohydride (${\mathrm{NaBH}}_{4}$) from Thermo Scientific Chemicals (Waltham, MA, USA), and methyl orange powder ($C_{14}H_{14}N_{3}{\mathrm{NaO}}_{3}S$) from LabChem (Auckland, New Zealand) were used as obtained. Sodium hydroxide (NaOH) from Ajax Finechem (New South Wales, Australia) was used as received. Deionized water with a resistance of 18 MΩ, taken from Scientific Promotion (Bangkok, Thailand) was used to prepare all aqueous solutions. All glasswares were cleaned with nitric acid, and then rinsed with deionized water prior to use.

### 3.2. Preparation of Nypa Fruticans Fruit Husk Extract and Green Synthesis of Ag NPs

The preparation of *Nypa fruticans* fruit husk (NF) extract and the synthesis of Ag NPs were carried out following our previous study [12]. Fresh NF husks were collected from the Pak Phanang River Basin, Nakhon Si Thammarat, Thailand. The husks were thoroughly washed with water, oven-dried at 60 °C for 42 h, and ground into a fine powder using an electric grinder (Philips Mixer Grinder HL1605). To obtain the extract, 10 g of NF powder was mixed with 100 mL of methanol and stirred continuously for 3 h. The mixture was then filtered through Whatman filter paper No. 1 (St. Louis, MO, USA), and the filtrate was concentrated using a vacuum evaporator at 50 °C. The obtained brown extract powder was stored at 4 °C for further applications.

For the green synthesis of Ag NPs, the method employed in our previous study was also used in the present study. In brief, a solution of 1.0 mL of 0.2% *w*/*v* NF extract was added dropwise to 20 mL of 1.0 mM silver nitrate under vigorous stirring at room temperature. The pH of the solution was adjusted to 9 using sodium hydroxide, and the reaction mixture was heated at 60 °C for 45 min. The formation of Ag NPs was confirmed by a color change to yellow, indicating the presence of reduced silver ions. It is noteworthy that the Ag NPs synthesized in this work were freshly prepared using the same synthesis method as in our previous study [12]. 

### 3.3. Characterization of Ag NPs 

The Ag NP colloidal solution was analyzed using a SPECTROstar Nano spectrophotometer (BMG LABTECH, Ortenberg, Germany) within the wavelength range of 300–800 nm to determine peak absorbance and characteristic surface plasmon resonance (SPR). The morphology and size of the nanoparticles were examined using Transmission Electron Microscopy (TEM; Talos F200i instrument, Thermo Fisher Scientific, Brno, Czech Republic) and Scanning Electron Microscopy (SEM; Apreo, FEI, Thermo Fisher Scientific, Brno, Czech Republic). Energy-Dispersive X-ray Spectroscopy (EDX; X-Max, Oxford. Instrument, UK) was utilized to confirm the formation of Ag NPs. Additionally, Fourier Transform Infrared Spectroscopy (FT-IR; INVENIO-S, Bruker, Germany) was performed to identify the functional groups present in both the NF extract and the synthesized Ag NPs.

### 3.4. Catalytic Tests for Methyl Orange Dye Degradation

The catalytic activity of the synthesized Ag NPs was evaluated using the reduction of methyl orange, a model azo dye, in the presence of ${\mathrm{NaBH}}_{4}$. In each test, 2.5 mL of 0.1 mM MO solution was mixed with 0.5 mL of 0.2 M ${\mathrm{NaBH}}_{4}$, followed by the addition of varying volumes (25–100 μL) of the Ag NP solution. The MO solution was prepared using deionized water. For the commercial synthetic dye, solutions were prepared using either deionized water or real water samples, non-potable tap water collected at Nakhon Si Thammarat Rajabhat University (two independent samples from different locations; no pH adjustment) prior to the degradation tests. The degradation process was monitored by recording the UV–Vis absorption spectra of the reaction mixture in the wavelength range of 200–700 nm at regular time intervals, until the characteristic orange color of MO completely faded, indicated by the disappearance of the peak at 465 nm. The obtained data were used to analyze the degradation kinetics. Control experiments under identical conditions but without the addition of Ag NPs were also conducted to confirm that the observed dye degradation was attributed to the catalytic activity of the Ag NPs.

### 3.5. Quantum Chemical Calculations 

All quantum chemical calculations were performed using the Gaussian 09 package [65]. Geometry optimizations for methyl orange (MO) and other relevant intermediates and transition states were conducted using the B3LYP functional [66,67] with the 6-31++G(d,p) basis set. In what follows, the MO molecule without the ${\mathrm{Na}}^{+}$ counterion is denoted as ${\mathrm{MO}}^{-}$. Electronic self-consistency used the following convergence criteria: root mean square (RMS) density matrix change of 1.0 × 10^−8^, maximum density matrix change of 1.0 × 10^−6^, and energy change of 1.0 × 10^−6^ Hartree. Geometry optimizations were iterated until the default force and displacement thresholds were satisfied as follows: maximum force of 4.5 × 10^−4^ Hartree/Bohr, RMS force of 3.0 × 10^−4^ Hartree/Bohr, maximum displacement of 1.8 × 10^−3^ Bohr, and RMS displacement of 1.2 × 10^−3^ Bohr. Frequency calculations were performed to obtain free-energy corrections and to confirm each stationary point as either an intermediate (no imaginary frequencies) or a transition state (a single imaginary frequency) [68]. 

Subsequently, single-point calculations were conducted at a higher level of theory, B3LYP-D3/6-311++G(d,p), using the polarizable continuum model (PCM) to mimic solvation in water. The D3 correction by Grimme et al. [69] was included to account for electronic dispersion interactions. Partial atomic charges were derived from the natural population analysis (NPA) [70]. Condensed Fukui functions were computed based on Hirshfeld charges, which have been demonstrated as effective tools for identifying reactive sites [59,60,71,72]. Moreover, time-dependent DFT (TD-DFT) calculations were performed to predict the UV–Vis spectra, employing the same functional, basis set, and PCM solvation model as in the single-point calculations.

The optimized gas-phase structures were subjected to restrained electrostatic potential (RESP) fitting to determine partial atomic charges. To maintain consistency with the standard General Amber Force Field (GAFF) approach [73] used in the molecular dynamics simulations, assessment of the partial charges is based on single-point calculations at the HF/6-31+G(d) level. 

### 3.6. Molecular Dynamics Simulations

Molecular dynamics (MD) simulations were conducted with the Large-scale Atomic/Molecular Massively Parallel Simulator (LAMMPS) software package (23 Jun 2022—Update 4) [74], utilizing a 1 fs time step for numerical integration. For bulk phase simulations, shifted-force potentials with a 12 Å cutoff were applied. To approximate Ewald summation for the long-range Coulomb interactions, the damped-shifted force potential with a damping parameter of 0.05 Å^−1^ was employed [75]. The simulations were carried out in the canonical (NVT) ensemble with a Nosé–Hoover thermostat to control the temperature.

A slab model of the Ag(111) surface, as used in our previous study [12], was constructed using the VESTA program [76]. The unit cell was replicated 6 × 11 × 3 times, resulting in a slab size of 30.0 × 31.8 × 21.2 Å^3^. During the MD simulations, we fixed the three bottom layers and applied 2D periodic boundary conditions to the Ag slab, with free boundary conditions in the [111] direction.

For modeling the Ag–water interface, a film of 2000 $H_{2}O$ molecules placed on the Ag slab was employed in analogy to a previous study of heavy metal association with Ag(111) [12]. Prior to the production runs, the system containing 2 ${\mathrm{Na}}^{+}$, 1 ${\mathrm{BH}}_{4}^{-}$, and 1 ${\mathrm{MO}}^{-}$ in 2000 $H_{2}O$ was carefully relaxed to accommodate the interface with the Ag(111) slab. Initial energy minimization was performed using the conjugate gradient (CG) algorithm [77]. This was followed by a 4 ns run at 300 K in the NVT ensemble. Next, additional 4 ${\mathrm{Na}}^{+}$ and 4 ${\mathrm{BH}}_{4}^{-}$ ions were randomly introduced into the final configuration, ensuring a minimum distance of 0.5 Å from all existing atoms. Another round of CG minimization was performed, followed by a 24 ns simulation at 300 K, with structural properties sampled from the last 4 ns.

To further illustrate the validity of the water model, we computed the number density profile of $H_{2}O$ along the surface normal (Figure S3). In the bulk-like region of the slab, the density reached a stable plateau of ~0.10 atoms/Å^3^, equivalent to ~0.033 molecules/Å^3^, which converts to ~0.996 g/cm^3^. This value is in excellent agreement with the experimental bulk water density (0.9965 g/cm^3^ at 298 K). As expected, oscillations were observed near the Ag surface due to interfacial layering, while a gradual density decay occurred at the liquid–vacuum interface [78,79]. 

The Ag-Ag interaction potential for the silver metal was taken from the Embedded Atom Model (EAM) [80]. In turn, interactions with C, O, and H atoms were described using GAFF2 [73] force fields. For this, the Lennard-Jones parameters for Ag were taken from the study by Heinz et al. [81], while those for ${\mathrm{Na}}^{+}$ were from Mamatkulov et al.’s study [82]. For mixing the van der Waals parameters, the Lorentz–Berthelot combination rules were applied.

## 4. Conclusions

This study demonstrates the application of Ag NPs green-synthesized using *Nypa fruticans* fruit husk extract as a reducing and stabilizing agent. These Ag NPs were well-dispersed with an average diameter of approximately 4 nm, as revealed by TEM. The presence of biomolecules from the extract, notably hydroxyl (–OH) groups, on Ag NPs was confirmed by FT-IR spectroscopy. Under the optimal synthesis condition (pH 9), surface groups are predominantly deprotonated, imparting a negative surface charge on the Ag NPs, as previously reported [12], which prevents uncontrolled aggregation.

Prior to their application in dye degradation, reaction parameters were systematically optimized. Our synthesized Ag NPs efficiently catalyzed the degradation of methyl orange dye in the presence of $\mathrm{Na}{\mathrm{BH}}_{4}$, achieving complete degradation within one minute. UV–Vis spectral analysis showed that the degradation process followed pseudo-first-order kinetics, with high rate constants indicating efficient catalytic activity.

The mechanism of MO degradation was elucidated through a combined approach of MD and DFT calculations, providing novel insights into the catalytic process. MD simulations suggested preferential adsorption of MO via its azo group onto the Ag(111) surface and the proximity of ${\mathrm{Na}}^{+}$ ions near the sulfonyl groups. DFT calculations, incorporating condensed Fukui function analysis, revealed that the initial step involves direct hydride transfer from ${\mathrm{BH}}_{4}^{-}$ to the N atom of the azo group via a low-energy transition state (TS1), which is more favorable than the ${\mathrm{Na}}^{+}$-mediated pathway (TS2). This first step represents the rate-determining process under neutral and acidic conditions. The subsequent step of MO degradation was also evaluated theoretically, revealing pH-dependent energy profiles due to proton involvement. Under alkaline conditions (at basic pH, >9), the second hydride transfer step becomes the rate-determining step, potentially reducing the degradation efficiency. These findings are consistent with previous experimental reports on the pH-dependent kinetics of MO degradation and provide additional mechanistic insight into the catalytic process. Further systematic experimental investigations are recommended to confirm the simulation-predicted effects of pH on the catalytic performance of Ag NPs.

Overall, this study highlights the potential of *Nypa fruticans* fruit husk extract for the sustainable synthesis of Ag NPs with excellent catalytic performance in dye degradation. Moreover, the theoretical insights gained from MD and DFT analyses complement the experimental results, offering a comprehensive understanding of the degradation mechanism and the influence of pH on the process. These insights could guide the design of more efficient catalytic systems for environmental remediation applications.

## Figures and Tables

**Table 1:** Summary of the optimized parameters for the synthesis of Ag NPs using NF extract [12].

Condition	Optimized Parameter
pH	9
Concentration of Ag${\mathrm{NO}}_{3}$	1.0 mM
Concentration of NF extract	0.2% *w*/*v*
Volume ratio of NF extract: Ag${\mathrm{NO}}_{3}$	1:20
Temperature	60 °C
Reaction time	45 min

**Table 2:** A summary of the percent of MO degradation by the synthesized Ag NPs in the presence of 0.05, 0.10, 0.15, 0.20, 0.25, and 0.30 M ${\mathrm{NaBH}}_{4}$, along with the corresponding pseudo-first-order kinetics parameters.

${\mathrm{NaBH}}_{4}$, (M)	% Degradation	R^2^	k (min^−1^)	Time (min)
0.05	95.99	0.8533	0.030995	6.5
0.10	95.55	0.9487	0.686816	4.5
0.15	95.33	0.9762	0.929336	4.5
0.20	99.50	0.9961	1.502668	2.5
0.25	99.66	0.9824	2.457128	2.5
0.30	99.03	0.9806	1.912067	2.5

**Table 3:** A summary of the percent of MO degradation and pseudo-first-order kinetics parameters for the different volumes of Ag NP catalysts ranging from 25 μL to 100 μL (see Figure 4e,f).

Volume of Ag NPs (μL)	% Degradation	R^2^	k (min^−1^)	Time (min)
25	95.36	0.9929	0.523583	4.5
50	95.73	0.9963	1.405366	1.5
75	95.58	0.9925	2.404223	1.0
100	99.26	0.9905	2.763169	1.0

**Table 4:** Comparison of the results of the catalytic MO degradation in the presence of ${\mathrm{NaBH}}_{4}$ with other studies. All the collected studies presented only focus on the green synthesis of Ag NPs using biomolecules from natural extracts.

Source of Ag NP Synthesis	Size (nm) ^a^	Degradation Percentage (%)	Reaction Rate (min^−1^)	Time (min)	[Ref.] (Year)
*Trigonella foenum-graecum* seeds	10–30	100 ^b^	0.6626	6	[20] (2014)
*Biophytum sensitivum*	19.06	100 ^b^	0.2758	9	[21] (2015)
*Punica granatum*	36	99	0.2175	12	[22] (2015)
*Anacardium occidentale* testa	25	100 ^b^	0.1178	20	[23] (2016)
*Sterculia acuminata* fruit	10	100 ^b^	0.0879	3	[24] (2016)
*Mussaenda erythrophylla* leaf	24–91	-	-	45	[25] (2016)
*Zanthoxylum armatum* leaves	15–50	-	0.00186	>24 h	[26] (2016)
*Durio zibethinus*	10–25	100 ^b^	0.636	7.5	[27] (2018)
*Medicago polymorpha*	25–33	97	0.348	3	[28] (2019)
*Euphorbia geniculata* leaf	17	97.28	-	30	[29] (2019)
*Centella asiatica*	30–50	84.38	-	180	[30] (2020)
*Prunus mume* (*P. mume*) fruit	30	99.96	0.0785	30	[31] (2020)
*Terminalia arjuna* leaf	10–50	86.68	0.166	14	[32] (2020)
*Calendula officinalis*	50–60	>95	0.18	10	[33] (2020)
*Dodonaea viscosa*	60	96.2	0.2925	4	[34] (2021)
*Eucalyptus globulus* fruit	20–100	>90%	0.247	10	[35] (2021)
*Sargassum serratifolium*	27.84	-	0.1580	16	[36] (2021)
*Simarouba glauca* oil seed meal	4.61	100 ^b^	0.055	40	[37] (2021)
*Bacillus cereus*	5–7.06	-	0.0976	>25	[38] (2021)
*Heterotheca subaxillaris* flower	20–30	100 ^b^	0.12	11	[39] (2022)
Neem	5–13	94.27	0.0857	35	[40] (2022)
*Rhus javanica*, *Rumex hastatus*, and *Callistemon viminalis*	55–67	80–83	-	120	[41] (2022)
*Sargassum horneri*	22.72	-	0.2266	22	[42] (2022)
Tea leaf and aloe vera leaf	90	100	0.500	12	[43] (2023)
*Klebsiella pneumoniae*	22.25–47.99	26.6	-	50	[44] (2024)
*Camellia sinensis*	74.85	93	0.087	40	[45] (2024)
*Usnea longissima* (Lichenan)	6.3	100 ^b^	1.481	2.67	[46] (2024)
*Trigonella foenum-graecum* L. seed (*HM 425*)	28	83.63	0.0412	39	[47] (2024)
*S. costus* root aqueous	22	72.88	0.0072	135	[48] (2024)
*Cymbopogon citratus* (lemongrass)	15–62.5	95.82	0.0413	90	[49] (2025)
*Ligustrum ovalifolium* flower	50–100	92	0.8344	8	[50] (2025)
*Morinda* *citrifolia* leaf	22.72	98.6	0.923	6	[51] (2025)
*Dipterocarpus retusus* branch	2.76	99.1	0.0692	14	[52] (2025)
*Annona reticulata* endophyte	175.2	95	-	15	[53] (2025)
*Paullinia cupana* Kunth leaf	39.33–126.2	96.42 ^c^	0.0946 ^c^	14 ^c^	[54] (2025)
*Zingiber sianginensis*	19.49	98	0.9918	3.67	[55] (2025)
*Nypa fruticans* fruit husk	4	>99	2.763	~1	This study

^a^ A single value indicates the reported average diameter size. ^b^ An exact value was not reported; however, we provide 100% degradation if the study stated a complete reduction of MO and the corresponding UV–Vis spectrum at the reduction time showed almost no absorption peak. ^c^ The results are based on the extract collected during the rainy season.

## Data Availability

Data are contained within the article or Supplementary Materials. Further inquiries can be directed to the corresponding author.

## Supplementary Materials

The following supporting information can be downloaded at: https://www.mdpi.com/article/10.3390/molecules30183738/s1, Figure S1: comparison of the UV–Vis spectra of the Ag NPs synthesized in this study (orange curve) and in our previous report (Wonglakhon et al., 2025 [12], blue curve). Both batches exhibit a characteristic SPR band at 424 nm. The peak absorbance at 424 nm is 1.6415 (this study) vs. 1.6493 (previous study), indicating < 0.5% difference; Figure S2: UV–Vis spectral changes for the reduction of the commercial synthetic dye by the synthesized Ag NPs in real water. Spectra were recorded in non-potable tap water from different locations: left, real water sample I; right, real water sample II. Legends indicate degradation time and the corresponding % degradation. For both samples, degradation reached ~83% at 5 min and ~86% by 6 min, accompanied by decolorization from deep yellow to nearly colorless, indicating a modest matrix effect while maintaining high catalytic activity; Figure S3: number density profile of water (in Å^−3^) along surface normal of the Ag(111) surface. A red dashed line marks the position where the number density reaches a plateau, indicating convergence. This distance was used as a reference point for normalizing the radial distribution functions; Figure S4: survival probability of a sodium ion within 5 Å of the oxygen atoms of the sulfonate group of MO dye. An exponential decay function was fitted to the data, providing an average residence time of 5.39 ps; Figure S5: relative free-energy profile (in kcal/mol) for the first step of MO degradation via (i) a direct hydride transfer (TS1), (ii) Na+-mediated hydride transfer (TS2), and (iii) direct hydride transfer with the incorporation of Ag atom (TS1-Ag)—from ${\mathrm{BH}}_{4}^{-}$ to N9 atom of the azo group. The 3D-optimized structures MO-Ag and TS1-Ag, with distance labels in Å.

## Abbreviations

The following abbreviations are used in this manuscript:

Ag NPs	Silver nanoparticles
SPR	Surface plasmon resonance
NF	*Nypa fruticans* fruit husk
Ag${\mathrm{NO}}_{3}$	Silver nitrate
$\mathrm{Na}{\mathrm{BH}}_{4}$	Sodium borohydride
NaOH	Sodium hydroxide
MO	Methyl orange
TEM	Transmission Electron Microscopy
SEM	Scanning Electron Microscopy
EDX	Energy-Dispersive X-ray Spectroscopy
FT-IR	Fourier Transform Infrared Spectroscopy
DFT	Density functional theory
TD-DFT	Time-dependent density functional theory
PCM	Polarizable continuum model
NPA	Natural population analysis
RMS	Root mean square
MD	Molecular dynamics
RDF	Radial distribution function
CG	Conjugate gradient
RESP	Restrained electrostatic potential
GAFF	General Amber Force Field
EAM	Embedded Atom Model
