# Supplementary material for: Green Synthesized Silver Nanoparticles from Biowaste for Rapid Dye Degradation: Experimental Investigation and Computational Mechanistic Insights
Source: Molecules. 2025 Sep 15;30(18):3738. doi: 10.3390/molecules30183738 (PMC12472237; doi:10.3390/molecules30183738)
Supplement: Supplementary file 1 [file molecules-30-03738-s001.zip › molecules-3842749-supplementary.pdf]

Green Synthesized Silver Nanoparticles from Biowaste for Rapid Dye Degradation: Experimental Investigation and Computational Mechanistic Insights

Tanakorn Wonglakhon[1,2], Areeya Chonsakon[3], Prawit Nuengmatcha[3], Benjawan Ninwong[3], Dirk Zahn[4], Yanisa Thepchuay[3,5]*

[1]Futuristic Science Research Center, School of Science, Walailak University, Nakhon Si Thammarat 80160, Thailand

[2]Research Center for Theoretical Simulation and Applied Research in Bioscience and Sensing, Walailak University, Nakhon Si Thammarat 80160, Thailand

[3]Center of Excellence in Nanomaterials Chemistry and Department of Chemistry, Faculty of Science and Technology, Nakhon Si Thammarat Rajabhat University, Nakhon Si Thammarat 80280, Thailand

[4]Lehrstuhl für Theoretische Chemie / Computer Chemie Centrum, Friedrich-Alexander-Universität Erlangen-Nürnberg, Nägelsbachstraße 25, 91052 Erlangen, Germany

[5]Flow Innovation-Research for Science and Technology Laboratories (*Firstlabs*), Bangkok 10400, Thailand

*Corresponding authors's email: yanisa_tep@nstru.ac.th

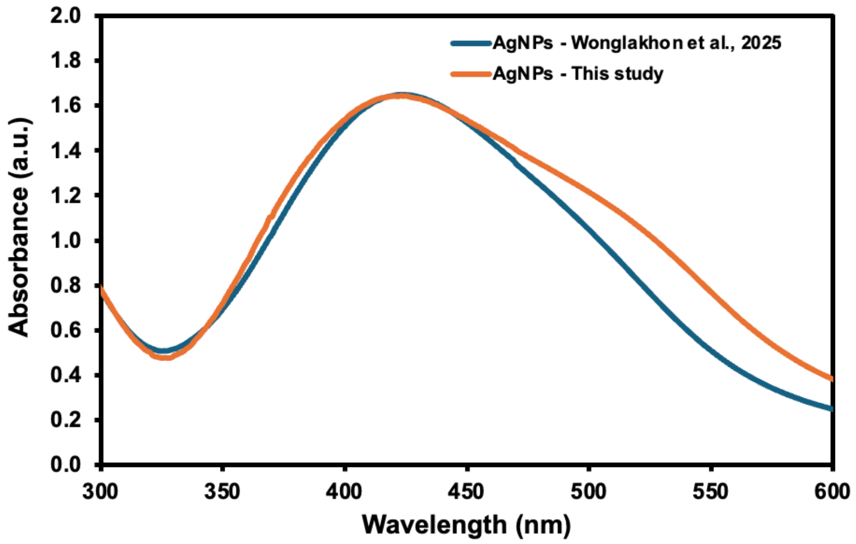

Figure S1. Comparison of the UV–Vis spectra of the Ag NPs synthesized in this study (orange curve) and in our previous report (Wonglakhon et al., 2025 [12], blue curve). Both batches exhibit a characteristic SPR band at 424 nm. The peak absorbance at 424 nm is 1.6415 (this study) vs. 1.6493 (previous study), indicating < 0.5% difference.

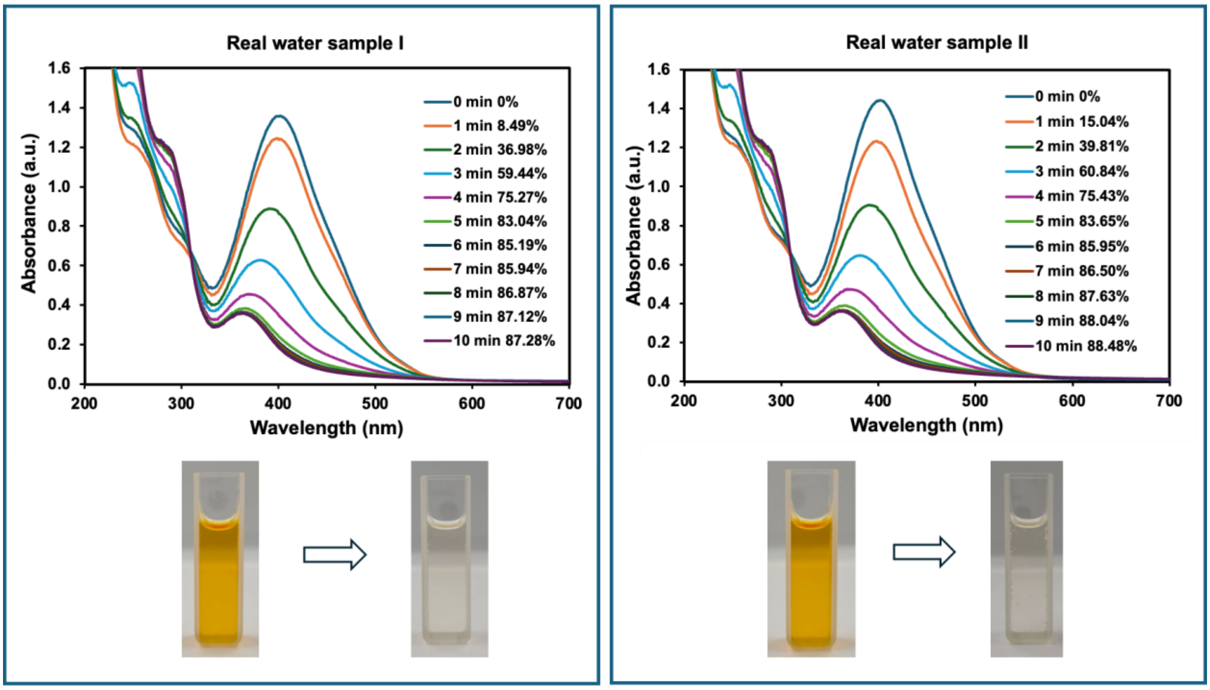

Figure S2. UV–Vis spectral changes for the reduction of the commercial synthetic dye by the synthesized Ag NPs in real water. Spectra were recorded in non-potable tap water from different locations: left, real water sample I; right, real water sample II. Legends indicate degradation time and the corresponding % degradation. For both samples, degradation reached ~83% at 5 min and ~86% by 6 min, accompanied by decolorization from deep yellow to nearly colorless, indicating a modest matrix effect while maintaining high catalytic activity.

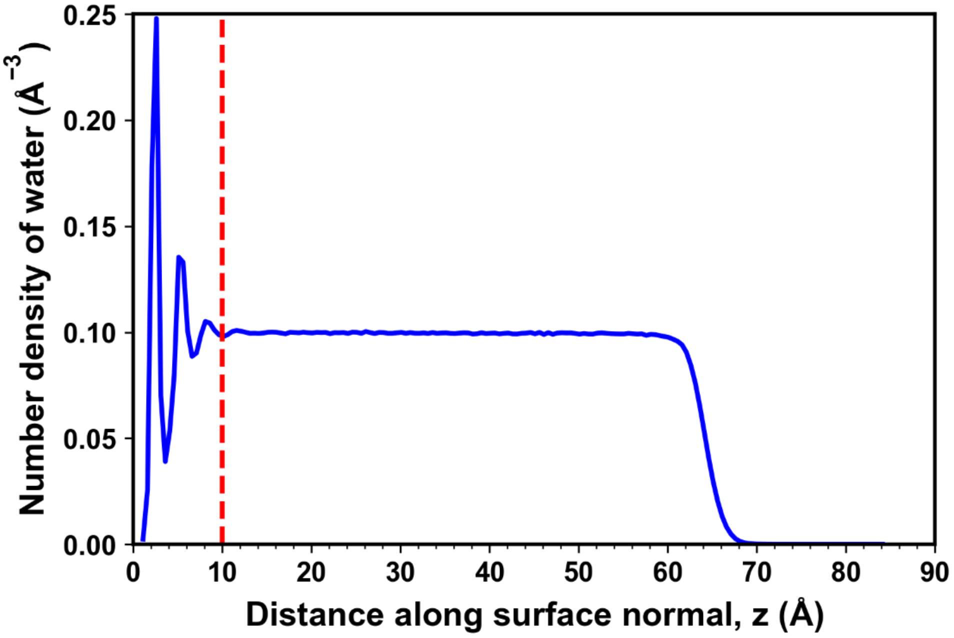

Figure S3. Number density profile of water (in Å^{-3}) along surface normal of the Ag(111) surface. A red dashed line marks the position where the number density reaches a plateau, indicating convergence. This distance was used as a reference point for normalizing the radial distribution functions.

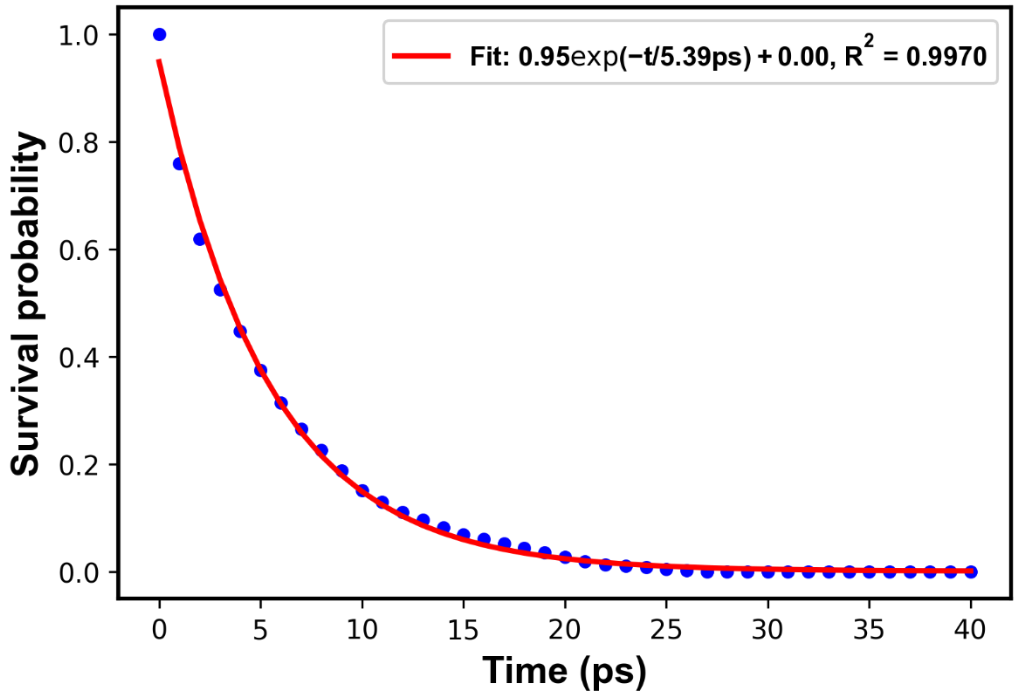

Figure S4. Survival probability of a sodium ion within 5 Å of the oxygen atoms of the sulfonate group of MO dye. An exponential decay function was fitted to the data, providing an average residence time of 5.39 ps.

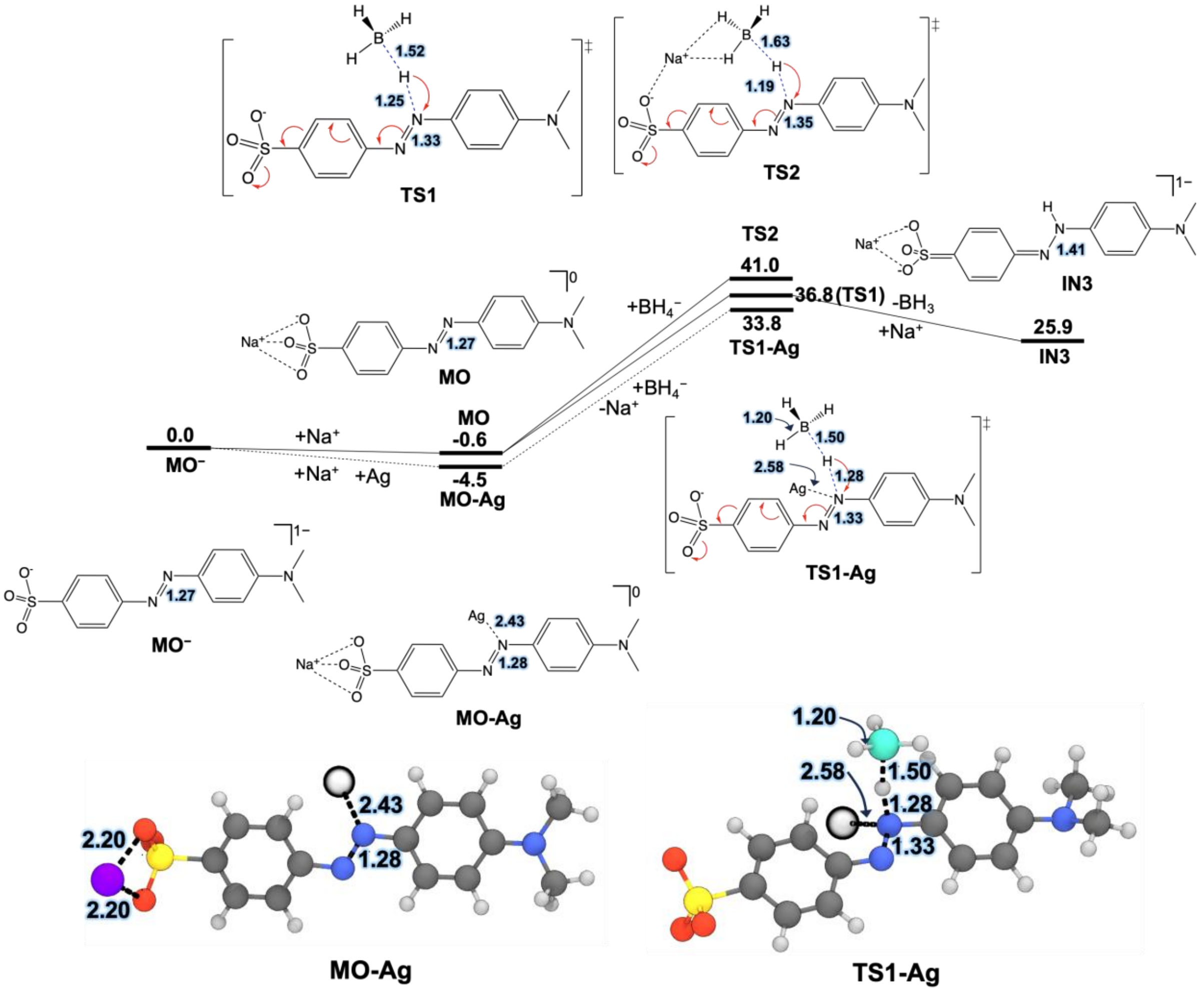

Figure S5. Relative free-energy profile (in kcal/mol) for the first step of MO degradation via (i) a direct hydride transfer (**TS1**), (ii) Na^{+}-mediated hydride transfer (**TS2**), and (iii) direct hydride transfer with the incorporation of Ag atom (**TS1-Ag**)–from BH_4^- to N9 atom of the azo group. The 3D-optimized structures **MO-Ag** and **TS1-Ag**, with distance labels in Å.

Cartesian coordinates

MO^-

H	-1.41645	-2.37862	0.02401
H	-3.93883	-2.20443	-0.03630
H	5.20535	2.19910	0.04790
H	7.28685	1.47311	-1.12401
H	2.75466	2.41245	0.02710
H	7.22832	1.83226	0.61388
C	-1.92061	-1.41626	0.00733
C	-3.30809	-1.32215	-0.01785
C	7.42951	1.04336	-0.11861
O	-6.20480	-1.21431	-0.59618
C	4.60016	1.30097	0.05888
C	3.21628	1.42975	0.04548
H	8.47884	0.76056	-0.01316
N	0.26368	-0.47621	0.02821
C	-1.12622	-0.25723	0.00522
C	-3.92234	-0.06704	-0.04270
S	-5.73962	0.06913	-0.00771
N	6.59665	-0.11882	0.13275
C	5.20741	0.02631	0.08380
O	-6.01597	1.27517	-0.83197
C	2.37568	0.31016	0.04652
N	0.98589	0.56536	0.02513
C	-1.74850	1.00791	-0.02937
C	-3.13178	1.09405	-0.05444
O	-6.04087	0.23046	1.43963
H	8.26252	-1.35548	-0.04798
C	7.17679	-1.42159	-0.14317
C	4.35144	-1.10257	0.07227
C	2.97273	-0.96208	0.05845
H	-1.12525	1.89533	-0.04206
H	6.94060	-1.79817	-1.15187
H	6.83054	-2.16575	0.58174
H	-3.63360	2.05492	-0.10456
H	4.76823	-2.10270	0.07266
H	2.32707	-1.83311	0.05071

MO

S	5.14177	-0.03620	0.00257
C	3.36396	0.11671	0.04775
C	2.78623	1.38643	0.03508
C	1.39879	1.49462	0.02595
C	0.59366	0.34662	0.02894
C	1.19326	-0.92629	0.04834
C	2.57611	-1.04080	0.05823
N	-0.80298	0.58326	0.01883
N	-1.51135	-0.46667	0.01897
C	-2.89752	-0.25596	0.00864
C	-3.70079	-1.40637	0.00531
C	-5.08377	-1.32291	-0.00500
C	-5.72998	-0.06185	-0.01213
C	-4.90910	1.09972	-0.00830
C	-3.53123	1.00198	0.00164
N	-7.10406	0.03884	-0.02238
C	-7.92256	-1.16215	-0.02531
C	-7.74470	1.34379	-0.02768
O	5.59805	-0.18394	-1.42383
O	5.73803	1.20122	0.60555
O	5.53071	-1.25628	0.78717
H	3.42081	2.26513	0.04682
H	0.91043	2.46355	0.02023
H	0.55650	-1.80279	0.06010
H	3.05549	-2.01307	0.08970
H	-3.20728	-2.37331	0.01085
H	-5.66458	-2.23643	-0.00737
H	-5.36145	2.08371	-0.01321
H	-2.91441	1.89349	0.00434
H	-7.74541	-1.77910	0.86570
H	-8.97534	-0.87951	-0.03398
H	-7.73214	-1.78355	-0.91044
H	-7.47036	1.93101	-0.91406
H	-8.82678	1.21153	-0.03564
H	-7.48346	1.93201	0.86204
Na	7.61907	-0.29006	-0.09401

TS1

B	-1.06459	3.42679	-0.83134
H	0.02979	3.56835	-1.31577
H	-0.90387	2.00406	-0.33427
H	-2.00181	3.31213	-1.58656
H	-1.28089	3.98987	0.22196
H	1.49636	-1.42111	-2.08651
H	3.96335	-1.56986	-1.66263
H	-4.42653	-2.19116	-0.47679

H	-6.70459	-2.53997	-0.37581
H	-2.05171	-1.52758	-0.75297
H	-6.09893	-2.51353	1.29726
C	1.96725	-0.83502	-1.30009
C	3.33179	-0.92234	-1.06149
C	-6.77109	-2.01136	0.59630
O	5.78191	-1.46405	1.29299
C	-4.12025	-1.18729	-0.19454
C	-2.77938	-0.82843	-0.36048
H	-7.80120	-2.12771	0.95796
N	-0.22006	-0.00836	-0.84024
C	1.13071	0.01055	-0.52926
C	3.93239	-0.15314	-0.05476
S	5.70150	-0.34148	0.31145
N	-6.44513	-0.60111	0.52956
C	-5.05403	-0.28338	0.32148
O	6.11643	0.97514	0.87853
C	-2.32958	0.46247	-0.03394
N	-0.95815	0.78406	-0.06015
C	1.75352	0.79938	0.46945
C	3.12730	0.71156	0.69351
O	6.32533	-0.66338	-1.00673
H	-8.38774	0.00624	0.00239
C	-7.35550	0.11219	-0.35833
C	-4.59685	1.00373	0.66463
C	-3.27463	1.37629	0.48028
H	1.14057	1.46666	1.06604
H	-7.32039	-0.25649	-1.40269
H	-7.10503	1.17437	-0.37393
H	3.60297	1.32679	1.45188
H	-5.30010	1.71489	1.09325
H	-2.94726	2.37874	0.73172

TS2

B	-0.02881	2.09826	0.83117
H	-0.39837	1.34607	1.70979
H	0.55323	0.96493	-0.18987
H	1.02264	2.64895	1.00055
H	-0.87187	2.70394	0.20732
H	-1.38936	-2.71168	0.83562
H	-3.85540	-2.27297	0.89828
H	4.99329	-2.11208	0.43477

H	6.97634	-1.19545	1.78049
H	2.54604	-2.09190	0.19935
H	7.08406	-2.08154	0.24594
C	-1.83187	-1.91253	0.24696
C	-3.19430	-1.67208	0.28143
C	7.29916	-1.12230	0.72747
O	-6.09889	-0.97486	0.71522
C	4.50626	-1.18836	0.14461
C	3.11948	-1.19230	0.01375
H	8.38334	-0.98651	0.70941
N	0.38298	-1.12329	-0.17395
C	-0.94834	-1.04104	-0.45435
C	-3.74822	-0.58982	-0.43460
S	-5.32577	0.11995	0.08945
Na	-2.61569	1.32072	1.07895
N	6.67291	-0.03058	0.00325
C	5.26559	-0.02526	-0.08958
O	-5.92636	0.76637	-1.09831
C	2.42857	-0.03393	-0.36185
N	1.03300	-0.03103	-0.62330
C	-1.54065	-0.03416	-1.27570
C	-2.92204	0.16805	-1.27034
O	-4.81969	1.13862	1.10891
H	8.43170	1.07695	0.13022
C	7.35361	1.24928	0.08175
C	4.55552	1.14206	-0.44706
C	3.17382	1.13121	-0.58905
H	-0.89898	0.56397	-1.91116
H	7.06043	1.85382	0.95782
H	7.16059	1.84645	-0.81503
H	-3.36780	0.96871	-1.85611
H	5.07916	2.07477	-0.61812
H	2.65760	2.04540	-0.86394

IN3

H	-1.19149	2.40376	0.22555
H	-3.62124	1.99763	0.45523
H	5.72111	-1.87679	-1.26973
H	7.52605	-0.47260	1.91971
H	3.26887	-2.12921	-1.38436
H	7.29337	-1.82882	0.79596
C	-1.58795	1.39179	0.20044
C	-2.93776	1.16395	0.31632
C	7.70760	-0.82195	0.88429

O	-5.84032	0.79319	0.91158
C	5.07860	-1.12693	-0.81336
C	3.70043	-1.26471	-0.88282
H	8.79375	-0.88225	0.73420
N	0.64335	0.65509	-0.12939
C	-0.63559	0.31912	0.04399
C	-3.45735	-0.15388	0.31176
S	-5.19090	-0.44811	0.28998
Na	-6.73672	1.37894	-0.96988
N	7.11480	0.04662	-0.12706
C	5.67533	-0.02344	-0.18020
O	-5.48604	-1.71173	0.99342
C	2.83887	-0.28593	-0.32851
N	1.47999	-0.45340	-0.35795
C	-1.19567	-1.01214	0.07696
C	-2.55718	-1.22587	0.19761
O	-5.66556	-0.47278	-1.17315
H	8.75951	1.32774	-0.25888
C	7.67878	1.38269	-0.07516
C	4.82334	0.94892	0.36421
C	3.43762	0.83810	0.28480
H	-0.52997	-1.86940	0.06786
H	7.54076	1.89882	0.89560
H	7.22675	2.00371	-0.85319
H	-2.94475	-2.24076	0.23272
H	5.24370	1.82229	0.85673
H	2.78275	1.60149	0.68571
H	1.14306	-1.14000	-1.03024

IN4

H	-1.77553	2.82698	-1.68595
H	-3.86716	1.57800	-1.19243
H	5.14616	0.04262	2.01564
H	6.96588	-0.98563	1.59311
H	3.32393	1.63970	2.18463
H	5.83818	-2.35593	1.68275
C	-1.76468	2.00260	-0.97685
C	-2.93565	1.30281	-0.71081
C	6.53199	-1.81191	1.01990
O	-5.53795	0.06760	-0.14789
C	4.54978	0.23319	1.13218
C	3.50282	1.14461	1.23241
H	7.34938	-2.48515	0.75467
N	0.60454	2.36436	-0.64372
C	-0.56513	1.66153	-0.32723
C	-2.92754	0.25582	0.21315
S	-4.41937	-0.67258	0.56530
Na	-6.02030	-1.68855	-1.39271
N	5.90554	-1.32434	-0.19665
C	4.82937	-0.43345	-0.07911
O	-4.54844	-0.76053	2.02577
C	2.68223	1.43000	0.13567
N	1.66815	2.41592	0.26111
C	-0.56714	0.60854	0.60439
C	-1.74393	-0.08378	0.87148
O	-4.26224	-2.01601	-0.12763
H	6.84030	-2.82838	-1.29244
C	5.94470	-2.20680	-1.34991
C	3.98196	-0.14808	-1.16993
C	2.93739	0.76669	-1.06725
H	0.35183	0.32280	1.10272
H	6.00899	-1.63109	-2.27928
H	5.06723	-2.87099	-1.42318
H	-1.74534	-0.88574	1.60210
H	4.12824	-0.64482	-2.12109
H	2.30235	0.96059	-1.92420
H	0.44627	3.26344	-1.08042
H	1.35701	2.53820	1.22279

IN5

H	-0.71582	1.65890	-1.20612
H	-3.15699	1.96742	-0.79641
H	5.58822	-2.09379	-0.12399
H	7.76951	-1.68930	0.49437
H	3.18035	-2.27543	0.20226
H	7.51702	-1.55631	-1.26392
C	-1.30703	0.85057	-0.78102
C	-2.66969	1.02931	-0.55489
C	7.82482	-1.00977	-0.36448
O	-5.67622	1.21025	-0.61029
C	5.01638	-1.18871	0.02819
C	3.65142	-1.29704	0.22048
H	8.86505	-0.71464	-0.49158
N	0.70098	-0.59690	-0.66920
C	-0.71003	-0.38069	-0.47802
C	-3.41652	-0.01394	-0.00294
S	-5.14142	0.27748	0.44851
Na	-7.32168	-0.21436	-1.15094

N	7.01162	0.18569	-0.15734
C	5.66553	0.08005	0.02874
O	-5.10159	0.81655	1.81131
C	2.89225	-0.14355	0.42900
N	1.44338	-0.25186	0.59746
C	-1.47108	-1.43497	0.04038
C	-2.82870	-1.24745	0.28734
O	-5.82528	-1.05807	0.28992
H	8.72386	1.36972	-0.31541
C	7.65518	1.49657	-0.15066
C	4.85445	1.23176	0.23327
C	3.48784	1.11857	0.42389
H	-1.00132	-2.39716	0.22299
H	7.51997	2.00728	0.81037
H	7.26516	2.14128	-0.94740
H	-3.43979	-2.04945	0.68624
H	5.29723	2.21809	0.24794
H	2.89670	2.01776	0.58318
H	1.09113	0.05639	-1.34978
H	1.19976	-1.00936	1.24365
H	1.06518	0.61438	1.00829

TS3

H	0.95427	-1.95892	0.67045
H	3.34469	-1.51901	1.28972
H	-5.58326	1.89310	-1.20253
H	-7.71333	1.91560	-0.63885
H	-3.17024	2.18917	-1.07225
H	-7.53447	0.62574	-1.84947
C	1.48260	-1.09414	0.28262
C	2.81331	-0.86402	0.60869
C	-7.82091	0.83790	-0.80922
O	5.72107	-0.75470	1.05192
C	-5.01770	1.24736	-0.54368
C	-3.64516	1.42089	-0.46797
H	-8.87564	0.58964	-0.68945
N	-0.52052	-0.38845	-0.98698
C	0.81875	-0.24189	-0.63032
C	3.48752	0.22162	0.03971
S	5.19900	0.55170	0.49261
Na	7.12599	-1.02695	-0.63373
N	-7.03977	0.07358	0.15018
C	-5.67226	0.24221	0.21173
O	5.19628	1.68275	1.42784
C	-2.86768	0.61043	0.36783
N	-1.46474	0.74060	0.38767
C	1.52548	0.84024	-1.20215
C	2.85104	1.07684	-0.86793
O	5.90645	0.80788	-0.82230
H	-8.73767	-1.02044	0.66427
C	-7.65912	-1.06174	0.81733
C	-4.86479	-0.58198	1.03544
C	-3.49359	-0.40269	1.10587
H	1.01041	1.46431	-1.92649
H	-7.47644	-1.03473	1.89801
H	-7.29423	-2.02458	0.43266
H	3.40295	1.89685	-1.31404
H	-5.30877	-1.38035	1.61510
H	-2.89956	-1.07111	1.72271
H	-0.90683	-1.26494	-0.59761
H	-1.11190	1.64051	0.08125
H	-1.01535	0.45123	1.25216
H	0.45303	-3.72394	-0.89335
B	-0.63802	-3.27915	-1.21917
H	-1.25438	-2.87355	-0.22056
H	-0.36945	-2.27992	-1.96165
H	-1.33399	-4.02333	-1.87836

P1

H	2.97890	-2.15383	0.01274
H	0.49744	-2.14486	-0.05126
C	2.43750	-1.21134	0.00677
C	1.04885	-1.21168	-0.02039
O	-1.90262	-1.23726	-0.71938
N	4.54204	-0.00004	0.10330
C	3.15430	-0.00001	0.02387
C	0.35442	0.00002	-0.03253
S	-1.42269	0.00003	-0.01549
O	-1.90262	1.23723	-0.71954
C	2.43754	1.21132	0.00678
C	1.04887	1.21170	-0.02039
O	-1.92676	0.00012	1.40446
H	2.97894	2.15381	0.01276
H	0.49751	2.14490	-0.05127
H	5.00179	-0.84025	-0.21744
H	5.00183	0.84014	-0.21746
Na	-3.91096	-0.00009	0.04797

P2

H	-0.41051	-2.15703	-0.06877
H	-2.46199	-2.02580	-0.60626
H	2.01527	-2.14623	0.03401
H	-2.57704	-1.60610	1.11648
C	-2.75184	-1.23408	0.09256
C	0.09564	-1.19965	-0.05660
C	1.48671	-1.19618	-0.00095
H	-3.82391	-1.06976	-0.03339
N	-2.04528	0.00002	-0.20327
C	-0.64377	-0.00003	-0.09950
C	2.21497	-0.00001	0.02463
N	3.61806	-0.00006	0.14775
H	-3.82367	1.07035	-0.03423
C	-2.75168	1.23415	0.09298
C	0.09561	1.19958	-0.05685
C	1.48669	1.19613	-0.00125
H	-2.46083	2.02630	-0.60491
H	-2.57772	1.60525	1.11738
H	-0.41056	2.15697	-0.06919
H	2.01521	2.14622	0.03337
H	4.05621	-0.82911	-0.23378
H	4.05610	0.82975	-0.23226

Reference:

[12] Wonglakhon, T.; Jommala, N.; Laksee, S.; Nuengmatcha, P.; Ninwong, B.; Zahn, D.; Thepchuay, Y. Experimental and Computational Study of Ecofriendly Synthesis of Silver Nanoparticles from Natural Extracts: Self-Controlled Nucleation and Growth, and Colorimetric Detection of Heavy Metal Ions. *Surf. Interfaces* **2025**, *68*, 106618. https://doi.org/10.1016/J.SURFIN.2025.106618.
